# Cardiomyocyte-Specific Ablation of Med1 Subunit of the Mediator Complex Causes Lethal Dilated Cardiomyopathy in Mice

**DOI:** 10.1371/journal.pone.0160755

**Published:** 2016-08-22

**Authors:** Yuzhi Jia, Hsiang-Chun Chang, Matthew J. Schipma, Jing Liu, Varsha Shete, Ning Liu, Tatsuya Sato, Edward B. Thorp, Philip M. Barger, Yi-Jun Zhu, Navin Viswakarma, Yashpal S. Kanwar, Hossein Ardehali, Bayar Thimmapaya, Janardan K. Reddy

**Affiliations:** 1 Department of Pathology, Feinberg School of Medicine, Northwestern University, Chicago, Illinois, United States of America; 2 Department of Medicine, Feinberg School of Medicine, Northwestern University, Chicago, Illinois, United States of America; 3 Next Generation Sequencing Core Facility, Feinberg School of Medicine, Northwestern University, Chicago, Illinois, United States of America; 4 Center for Cardiovascular Research, Washington University School of Medicine, St. Louis, Missouri, United States of America; 5 Department of Surgery, University of Illinois at Chicago, Chicago, Illinois, United States of America; 6 Department of Microbiology and Immunology, Feinberg School of Medicine, Northwestern University, Chicago, Illinois, United States of America; University of Florida, UNITED STATES

## Abstract

Mediator, an evolutionarily conserved multi-protein complex consisting of about 30 subunits, is a key component of the polymerase II mediated gene transcription. Germline deletion of the Mediator subunit 1 (Med1) of the Mediator in mice results in mid-gestational embryonic lethality with developmental impairment of multiple organs including heart. Here we show that cardiomyocyte-specific deletion of Med1 in mice (csMed1^-/-^) during late gestational and early postnatal development by intercrossing Med1^fl/fl^ mice to α-MyHC-Cre transgenic mice results in lethality within 10 days after weaning due to dilated cardiomyopathy-related ventricular dilation and heart failure. The csMed1^-/-^ mouse heart manifests mitochondrial damage, increased apoptosis and interstitial fibrosis. Global gene expression analysis revealed that loss of Med1 in heart down-regulates more than 200 genes including *Acadm*, *Cacna1s*, *Atp2a2*, *Ryr2*, *Pde1c*, *Pln*, *PGC1α*, and *PGC1β* that are critical for calcium signaling, cardiac muscle contraction, arrhythmogenic right ventricular cardiomyopathy, dilated cardiomyopathy and peroxisome proliferator-activated receptor regulated energy metabolism. Many genes essential for oxidative phosphorylation and proper mitochondrial function such as genes coding for the succinate dehydrogenase subunits of the mitochondrial complex II are also down-regulated in csMed1^-/-^ heart contributing to myocardial injury. Data also showed up-regulation of about 180 genes including *Tgfb2*, *Ace*, *Atf3*, *Ctgf*, *Angpt14*, *Col9a2*, *Wisp2*, *Nppa*, *Nppb*, *and Actn1* that are linked to cardiac muscle contraction, cardiac hypertrophy, cardiac fibrosis and myocardial injury. Furthermore, we demonstrate that cardiac specific deletion of Med1 in adult mice using tamoxifen-inducible Cre approach (TmcsMed1^-/-^), results in rapid development of cardiomyopathy and death within 4 weeks. We found that the key findings of the csMed1^-/-^ studies described above are highly reproducible in TmcsMed1^-/-^ mouse heart. Collectively, these observations suggest that Med1 plays a critical role in the maintenance of heart function impacting on multiple metabolic, compensatory and reparative pathways with a likely therapeutic potential in the management of heart failure.

## Introduction

Mediator, an evolutionarily conserved multi-protein complex consisting of about 30 subunits, is a major component of eukaryotic transcription machinery [1,2]. Mediator serves as a transcriptional nexus by integrating diverse signaling pathways through its interactions with sequence specific transcription factors, transcriptional coactivators, proteins that induce epigenetic alterations and RNA polymerase II [1,3,4]. Emerging knowledge indicates that different subunits of the Mediator have the potential to influence tissue specific gene expression programs and deletion of these subunits individually or mutations in one or more of these subunits can induce different disease conditions including cardiovascular diseases [5–9].

Med1, a major subunit of the Mediator, plays a central role in nuclear receptor mediated gene expression including peroxisome proliferator-activated receptor (PPAR)α-induced activation of an array of genes related to energy homeostasis in liver and ligand bound thyroid hormone receptor induced regulation of a large number of genes involved in protein, fat and carbohydrate metabolism [9–12]. Earlier, we and others have shown that germline deletion of Med1 is embryonically lethal with death occurring at embryonic day 11.5 (E11.5) [13–15]. The major cause of death of Med1 null mouse embryos at E11.5 was likely to be due to severe cardiac failure because of non-compaction of the ventricular myocardium and the resultant ventricular dilatation [16]. These poorly developed hearts showed large pericardial effusion associated with a dilated blood filled ventricle. These observations suggested that impaired signaling at the endocardial-myocardial junction may account for the loss of myocytes contributing to ventricular hypoplasia [16]. Because the above studies were carried out using a mouse model in which Med1 was deleted at the global level, it was not possible to delineate the role(s) played by Med1 in cardiac specific functions.

While the Mediator complex is expected to play indispensable role in cardiac specific gene expression, no mutations in the Mediator complex subunits were reported in human hearts that are relevant to cardiac diseases. A large number of inherited mutations were shown to contribute to different forms of cardiomyopathy including dilated cardiomyopathy (DCM), arrhythmogenic right ventricular cardiomyopathy (ARVC), restrictive and hypertrophic cardiomyopathy [17]. However, these mutations do not map to any of the known members of the Mediator subunits. It is likely that any cardiac specific mutations in the Mediator subunits that are critical for its function in humans would be deleterious. Therefore to begin to understand the role of Med1 in myocardial function, we have now first generated Cre-loxP-mediated cardiomyocyte-specific deletion of Med1 in mice (csMed1^-/-^) by intercrossing Med1^fl/fl^ mice to α-MyHC-Cre transgenic mice that express Cre under the control of the α-myosin heavy chain (α-MyHC) gene promoter [18]. The csMed1^-/-^ mice die within 10 days after weaning due to dilated cardiomyopathy (DCM)-related ventricular dilatation and heart failure. Gene expression analysis indicated that loss of Med1 decreased expression of most of the genes involved in calcium signaling, cardiac muscle contraction and mitochondrial metabolic functions. The gene expression profile also showed a concurrent increase in the expression of genes involved in reparative cardiac function, including myocardial fibrosis. Our results suggest that the effects of abrogation of Med1 expression in heart may be biphasic in that reduction of genes critical for normal heart functions results in myocardial damage and heart failure and consequently genes related to metabolic pathways leading to cardiac injury, and cardiac fibrosis are elevated. Furthermore, to examine the role of Med1 in the adult mouse heart, we generated Tamoxifen inducible Cre mouse (TmcsMed1^-/-^). Using this mouse, we demonstrate that the key findings of the csMed1^-/-^ mouse studies described above are highly reproducible in TmcsMed1^-/-^ mouse heart. Overall, our studies for the first time provide evidence for a critical role for Med1 in heart function impacting on multiple pathways related to cardiac contraction, oxidative phosphorylation and energy metabolism.

## Experimental Procedures

### Generation of mice with cardiomyocyte-specific disruption of Med1 and ethics statement

A detailed description of the generation of the lox targeted Med1 allele has been published [19]. Med1^fl/fl^ mice were crossed with cardiac α-MyHC-Cre transgenic mice [18] to generate mice with cardiomyocyte-specific Med1 gene disruption (csMed1^-/-^) beginning during late embryonic period. PCR genotyping of mice was performed using the primers 5′-TCCATCTGACCTGCTGGATGATAA-3′ and 5′-GGGTGTGACCCCATAATT-3′ flanking *loxP* site 2 in the floxed Med1 allele [19]. *Cre*-specific primers used included: 5′-AGGTGTAGAGAAGGCACTCAGC-3′ and 5′-CTAATCGCCATCTTCCAGCAGG-3′. To generate mice with tamoxifen-inducible heart specific Med1 deletion (TmcsMed1^-/-^), Med1^fl/fl^ mice were crossed with Myh6-MCM (tamoxifen-inducible heart specific Cre) transgenic mice purchased from the Jackson Laboratory [12,20]. Seven-week old TmcsMed1^-/-^ mice and the wild-type littermates were then administered tamoxifen intraperitoneally at a daily dose of 65 mg/kg body weight for 5 days and then killed at selected intervals after initiation of tamoxifen treatment. For each experiment 3 to 5 mice for control and csMed1^-/-^ were used. To obtain survival curve 41 csMed1^-/-^ and 41 csMed1^fl/fl^ mice were used. Thirteen TmcsMed1^-/-^mice and the same number of littermates were used for the survival curve experiments using tamoxifen inducible model. The specific criteria for animal euthanasia included absence of food or water intake, slow or no mobility, weak or absence of heart beat, absence of palpitation of the chest as well as absence of respiratory movement. Mice were euthanized by intraperitoneal pentobarbital injection at the dose of 150mg/kg body weight to minimize suffering. Animals received food and water ad libitum and lighting was maintained on a 12-h light-dark cycle. All experimental procedures were performed in accordance with the National Institutes of Health Guide for Care and Use of Laboratory Animals. All animal studies were reviewed and approved by the Institutional Animal Care and Use Committee Northwestern University (protocol number 2013–3198).

### Echocardiography

Echocardiography was performed using a VisualSonics Vevo 770 high-resolution noninvasive transthoracic imaging system with a 30 MHz scanhead to monitor cardiac function [12]. Parasternal short- and long-axis views were used to obtain 2D and M-mode images. Long axis views facilitated examination of the septum, posterior wall and left ventricular outflow tract. Short axis views at the chordal level provided information on symmetry of wall thickness and contraction. At least 8 independent cardiac cycles per experiment were recorded.

### Histological analysis

Hearts from csMed1^-/-^ and Med1^fl/fl^ and also from TmcsMed1^-/-^ and the corresponding control mice were fixed in 4% paraformaldehyde overnight and processed for embedding in paraffin. Sections, 4-μm thick, were de-paraffinized and stained with hematoxylin and eosin (H&E). Immunohistochemical localization of Med1 was carried out using anti-MED1 antibody (catalog number sc-5334, Santa Cruz Biotechnology) as described previously [19]. Masson’s trichrome staining was used for the detection of cardiac fibrosis. Heart samples were also snap-frozen and sections ~ 5-μm thick were stained with Oil red O for the visualization of neutral lipid. To detect apoptosis, paraffin sections from Med1^fl/fl^ control, csMed1^-/-^ and TmcsMed1^-/-^ hearts, were processed for Terminal deoxynucleotidyl transferase (TdT) dUTP Nick-End Labeling (TUNEL), using an in situ detection kit according to the manufacturer’s instructions (Roche Diagnostics). Histological analysis and image processing were carried out using a Leica DMRE microscope equipped with Spot digital image analysis software and camera.

### Electron microscopy

For transmission electron microscopy, heart tissue collected from the left ventricle was fixed overnight with 3% glutaraldehyde in sodium cacodylate buffer at 4°C. After washing in cacodylate buffer for 1 h, the tissue was post-fixed in 1% osmium tetroxide in cacodylate buffer (pH 7.4) for 2 h at 4°C, and embedded in Epon [19]. Semi- and ultra-thin sections were cut with a Leica UC6 ultramicrotome, and examined with a FEI Tecnai Spirit transmission electron microscope.

### Library construction and RNA-sequencing

Library construction and sequencing were performed at the Genomics Core Facility at the University of Chicago. RNA quality and quantity were first determined with the Agilent Bioanalyzer 2100, accepting RNA integrity numbers (RIN) of >7 and quantities of 100 nanograms or more per sample. Samples were enriched for mRNA using oligo-dT columns. Directional 50 bp single-end mRNA libraries were prepared using Illumina TruSeq mRNA Sample Preparation Kits per manufacturer’s instructions (Illumina, San Diego, CA). Briefly, polyadenylated mRNAs were captured from total RNA using oligo-dT selection and converted to cDNA by reverse transcription. Each sample was ligated to Illumina sequencing adapters containing unique barcode sequences. Bar-coded samples were then amplified by PCR and the resulting cDNA libraries were quantified using qPCR. Finally, equimolar concentrations of each cDNA library were pooled and sequenced on the Illumina HiSeq2500.

### Transcriptome analysis

The quality of DNA reads in fastq format was evaluated using FastQC and the data were processed essentially as described [21]. Briefly, the reads were aligned to the *Mus musculus* genome (mm10) using TopHat (v2.0.8b). Subsequently, the aligned reads, in conjunction with a gene annotation file for mm10 obtained from the University of California, Santa Cruz (http://genome.ucsc.edu), were used to determine the expression of known genes using Cufflinks (v2.1.1). Differential expression was determined by cuffdiff using the procedure with an FDR cutoff value of 0.05 [21]. The results of the differential expression analysis were processed with cummeRbund. The differentially expressed genes were separated into those that were upregulated and those that were downregulated. A pathway analysis was performed on both gene lists using GeneCoDis [22–24] which identify pathways that are enriched with genes that are up-, and down-regulated.

### Quantitative real-time PCR

Total RNA was extracted from the csMed1^-/-^ and TmcMed1^-/-^ and the corresponding control mice TRIzol^®^ reagent (Lifetechnology, Carlsbad, CA). RNA was then purified using Qiagen RNeasy columns. cDNA was prepared with 2 μg of total RNA using SuperScript^™^ III First-Strand Synthesis System for RT–PCR (Invitrogen). Quantitative expression of specific genes was ascertained using SYBR Green (Life Technologies) in triplicates and normalized with 18S ribosomal RNA. Each PCR reaction contained of 1 μl (100 ρmol) of forward and reverse primers and 10 μl of 2×SYBR Green PCR Master Mix to make a final volume of 20 μl and the reaction was performed by using an ABI 7300 (Applied Biosystems). The relative gene expression changes were measured using the comparative *C*_*T*_ method, *X* = 2^−ΔΔ^*C*_*T*_.

### Mitochondrial DNA content

Mitochondrial DNA copy number was estimated as described [25]. Briefly, total DNA from heart tissue was isolated, then the quantity of the stable nuclear-encoded 18S ribosomal RNA (rRNA) and the mitochondrial encoded gene cytochrome c oxidase subunit 1 (CO1) were estimated by qPCR. Mitochondrial DNA copy number was expressed as the ratio of CO1 to 18S rRNA expression.

### Western blot analysis

Total proteins were extracted from the heart tissues of csMed1^-/-^ and TmcsMed1^-/-^ and the corresponding littermates and subjected to 4–20% SDS-PAGE and transferred to a nitrocellulose membrane (Invitrogen). Immunoblotting was performed using selected antibodies as described [19]. GAPDH was used as loading control. The protein bands were developed with an enhanced chemiluminescence substrate. Quantification of blots was performed using ImageJ software (NIH). Western blot analysis of Med1 was performed using crude nuclear extracts. Nuclei were prepared from the relevant heart tissues exactly as described [26]. Eight hearts were pooled from each group for nuclear isolation.

### Heart weight

Hearts from 25- to 27-day old csMed1^-/-^ and littermate controls were excised from anesthetized mice, transferred to ice-cold phosphate buffered saline and perfused essentially as described [27]. At the end of perfusion, the hearts were frozen in liquid nitrogen and stored at –80°C. Frozen heart was ground into powder and weighed (wet weight). The heart was then dried overnight and weighed to determine the dry weight.

### Statistical analysis

Student's *t* test was used to determine whether the sample was significantly different from the control. Differences were considered statistically significant at *P*<0.05, while *P*<0.01 represented more significant change.

## Results

### Generation of cardiomyocyte-specific Med1 heart knockout mice

**Fig 1:**
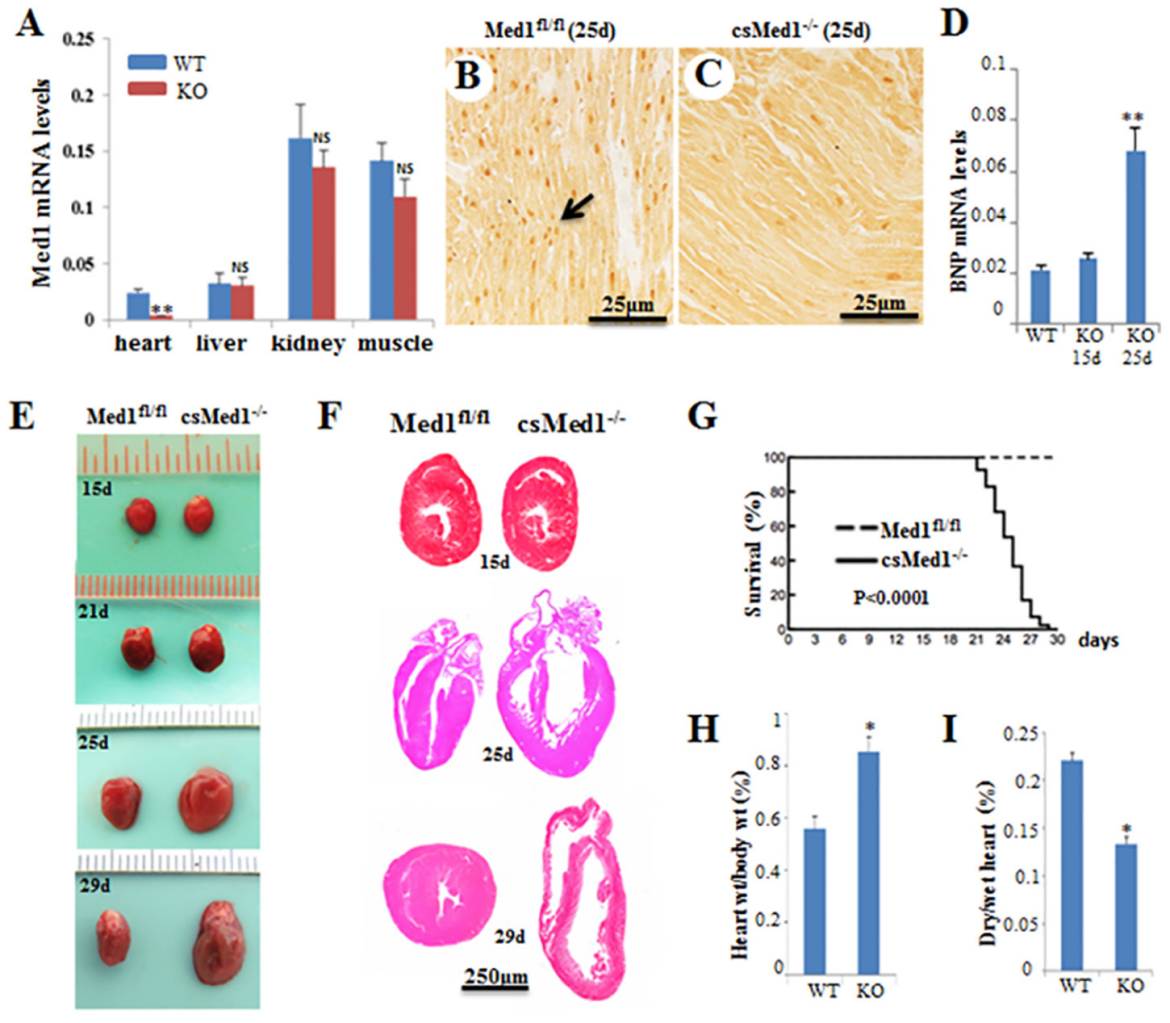
Cardiac-specific deletion of Med1 causes dilated cardiomyopathy. (A) Relative Med1 mRNA expression in Med1^fl/fl^ (WT) and csMed1^-/-^ mouse (KO) heart, liver, kidney and skeletal muscle. (B & C) Immunohistochemical localization of Med1 in 25-day old Med1 ^fl/fl^ (B, arrow), and csMed1^-/-^ (C) heart. (D) *Nppb* (BNP) mRNA level in 15-day old KO mouse heart is not increased as compared to 25-day old KO mouse. (E) Representative photographs of intact 15- day to-29 day-old csMed1^-/-^ mice and their corresponding Med1^fl/fl^ littermate. In 29-day old csMed1^-/-^ mice the heart was flaccid and flabby. (F) Heart sections of 15- day (upper pair), 25-day (middle pair) and 29- day old (lower pair) Med1 KO heart stained with H&E reveal thinning of ventricular walls and dilation of chambers. (G) Survival curve. 41 mice for each group of Med1^fl/fl^ and csMed1^-/-^ were used for the generation of survival curve. (H) Heart weight / body weight ratio. I, dry heart/wet heart ratio. Results, as indicated, are expressed as the mean ±SD. *p<0.05, **p<0.01, NS: non-significant.

Earlier studies showed that the globally targeted ablation of Med1/PBP/TRAP220 gene in mice results in embryonic lethality around gestational day 11.5 (E11.5) due to significant defects in the heart, eye, erythrocytes, megakaryocytes and vasculature [13–16]. To generate cardiomyocyte-specific Med1 gene disrupted mice (csMed1^-/-^), mice with a loxP flanked allele targeting exons 8–10 of Med1 (Med1^fl/fl^) [19] were crossed with α-MyHC-Cre transgenic mice that express Cre-recombinase in cardiomyocytes under the control of the α-myosin heavy chain (α-MyHC) gene promoter [18]. Disruption of the Med1 gene in cardiomyocytes was confirmed by PCR genotyping and by q-PCR analysis of RNA from mouse heart (Fig 1A). Med1 mRNA level was significantly decreased in csMed1^-/-^ mouse heart but not in liver and skeletal muscle (Fig 1A). Immunohistochemical localization of Med1 revealed prominent cardiomyocyte nuclear staining in the myocardium of Med1^fl/fl^ mice but not in the myocardium of csMed1^-/-^ littermates after weaning (Fig 1B and 1C). To more accurately determine the levels of Med1 in Med1 KO hearts, we have quantified the Med1 levels in TmcsMed1^-/-^ hearts using Western immunoblots (see below).

### Cardiomyocyte-specific disruption of Med1 causes dilated cardiomyopathy

**Fig 2:**
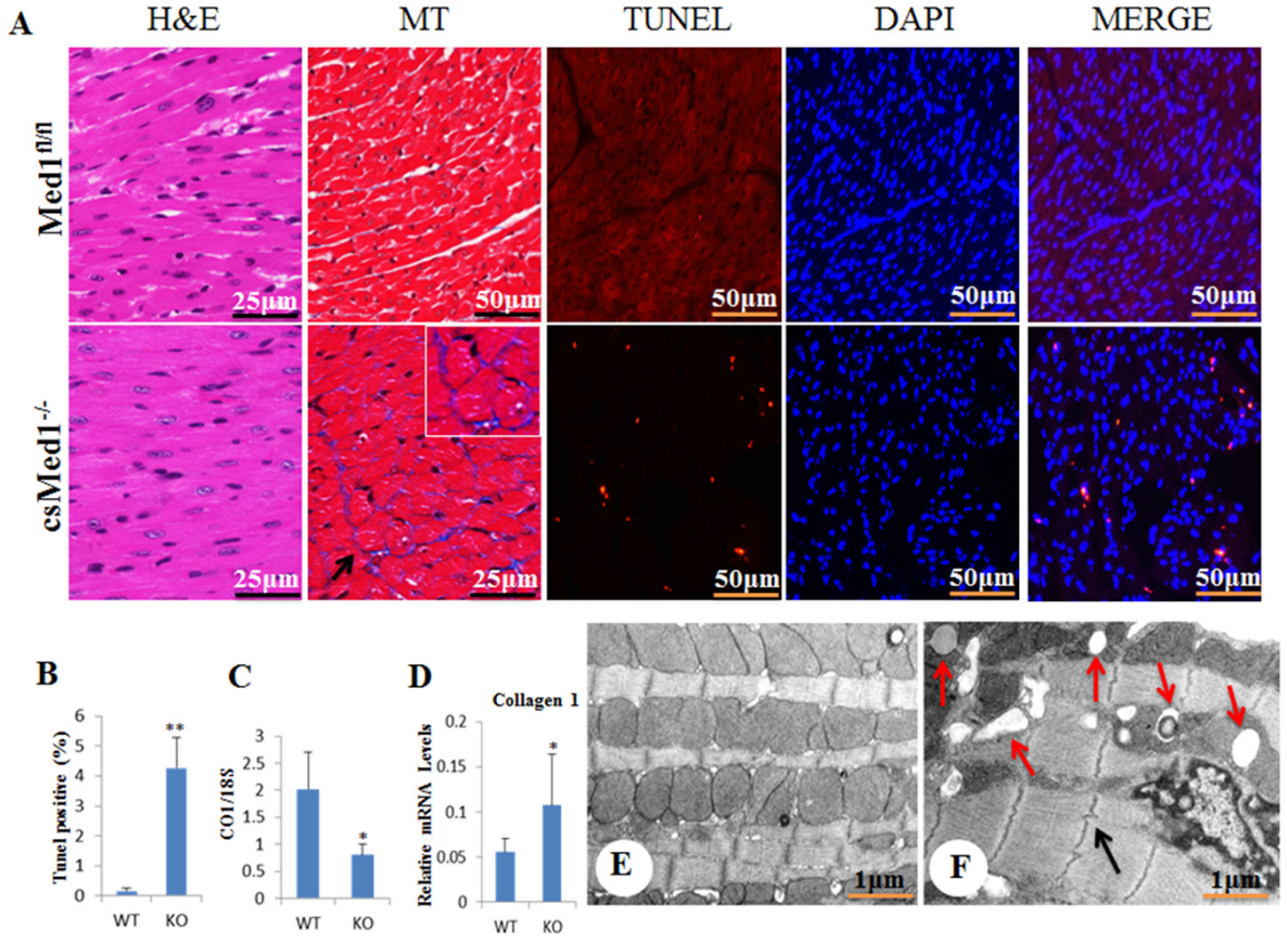
Cardiac fibrosis, increased cardiomyocyte apoptosis and mitochondrial damage in csMed1^-/-^ mouse heart. (A) Morphological changes in csMed1^-/-^ mouse heart as assessed by H&E, Masson’s trichrome (MT), TUNEL and DAPI staining. MERGE indicates DAPI and TUNEL overlap. (B) TUNEL positive cells in myocardium and (C) mitochondrial DNA content were determined by measuring the levels of the mitochondrial encoded gene cytochrome oxidase 1 (CO1) and the nuclear encoded 18S gene in Med1^fl/fl^ and csMed1^-/-^mouse heart. (D) qPCR data represent collagen mRNA transcript in Med1^fl/fl^ and csMed1^-/-^mouse heart. (E&F) Represent respectively the electron micrographs of 29-day old Med1^fl/f^ and csMed1^-/-^ mouse hearts. *Red arrows* in F indicate lipid droplets and electron dense degenerative bodies and *black arrow* points to irregularities in Z band pattern. Results are expressed as the mean ±SD. *p<0.05, **p<0.01.

csMed1^-/-^ mice were viable at birth with no gross morphological abnormalities. At two weeks after birth, there was no significant change in the heart size of the csMed1^-/-^ mice as compared to that of control mice (floxed littermates) (Fig 1E and 1F). We also tested the mRNA levels of heart failure indicators atrial natriuretic peptide (ANP/Nppa) and brain natriuretic peptide (BNP/Nppb). Both ANP and BNP levels increase in heart failure as ventricular cells are recruited to secrete both these peptides in response to left ventricular dysfunction [28]. As shown in Fig 1D, there was only a marginal increase in mRNA levels of these peptides in 15-day old csMed1^-/-^ mouse heart, which were not statistically significant. After, weaning the ANP and BNP levels were significantly increased in csMed1^-/-^ mouse heart. It is important to note that the cardiac myosin heavy chain gene expression undergoes a rapid transition from β- to α-myosin heavy chain during early rodent development and becomes exclusively expressed by postnatal day 21 [23]. Accordingly, the α-MyHC-Cre expression late in the embryonic development and maximally during the early postnatal period contributes to Med1 deletion resulting death of csMed1^-/-^ mice within ten days post weaning [23]. Thus, we believe that at birth csMed1^-/-^ possessed hearts with normal or near normal function. In contrast, in 22 to 29 day old csMed1^-/-^ mice, the size of the heart increased considerably when compared to littermate controls and the Med1 deficient hearts were increasingly flaccid (Fig 1E). Nearly 100% of csMed1^-/-^ mice died within 10 days after weaning due to dilated cardiomyopathy-related atrial and ventricular dilatation and heart failure (Fig 1E, 1F and 1G). Heart weight / body weight ratio increased in csMed1^-/-^ mice but dry heart weight was significantly decreased in these csMed1^-/-^ mice (Fig 1H and 1I), suggesting the edematous nature of csMed1^-/-^ mouse heart. In csMed1^-/-^ mice lung weight/body weight ratio was increased most likely due to pulmonary edema resulting from heart failure (data not shown). Histological examination of csMed1^-/-^ mouse hearts revealed dilatation of the right and left ventricular chambers with thinning of the walls and myocardial cells (Figs 1F and 2A). Masson trichrome staining showed evidence of interstitial myocardial fibrosis with increased collagen mRNA content in csMed1^-/-^ mouse hearts (Fig 2A). Evaluation of formalin fixed paraffin-embedded heart sections by TUNEL staining showed evidence of enhanced apoptosis in csMed1^-/-^ mouse hearts compared with that noted in normal hearts (Fig 2A and 2B). In the csMed1^-/-^ mouse heart the percentage of apoptotic cardiomyocytes increased significantly (Fig 2B). In csMed1^-/-^ mouse hearts the mitochondrial DNA content was decreased indicative of reduction in mitochondrial population (Fig 2C). Electron microscopic analysis of 23 to 29 day old csMed1^-/-^ mouse hearts demonstrated the presence of lipid vacuoles of differing sizes in cardiomyocytes and evidence of varying mitochondrial abnormalities (Fig 2E and 2F). Some mitochondria contained lipid droplets and membranous swirls (*red arrows*). Irregularities in Z band pattern were also noted (*black arrow*).

### Echocardiographic observations

**Fig 3:**
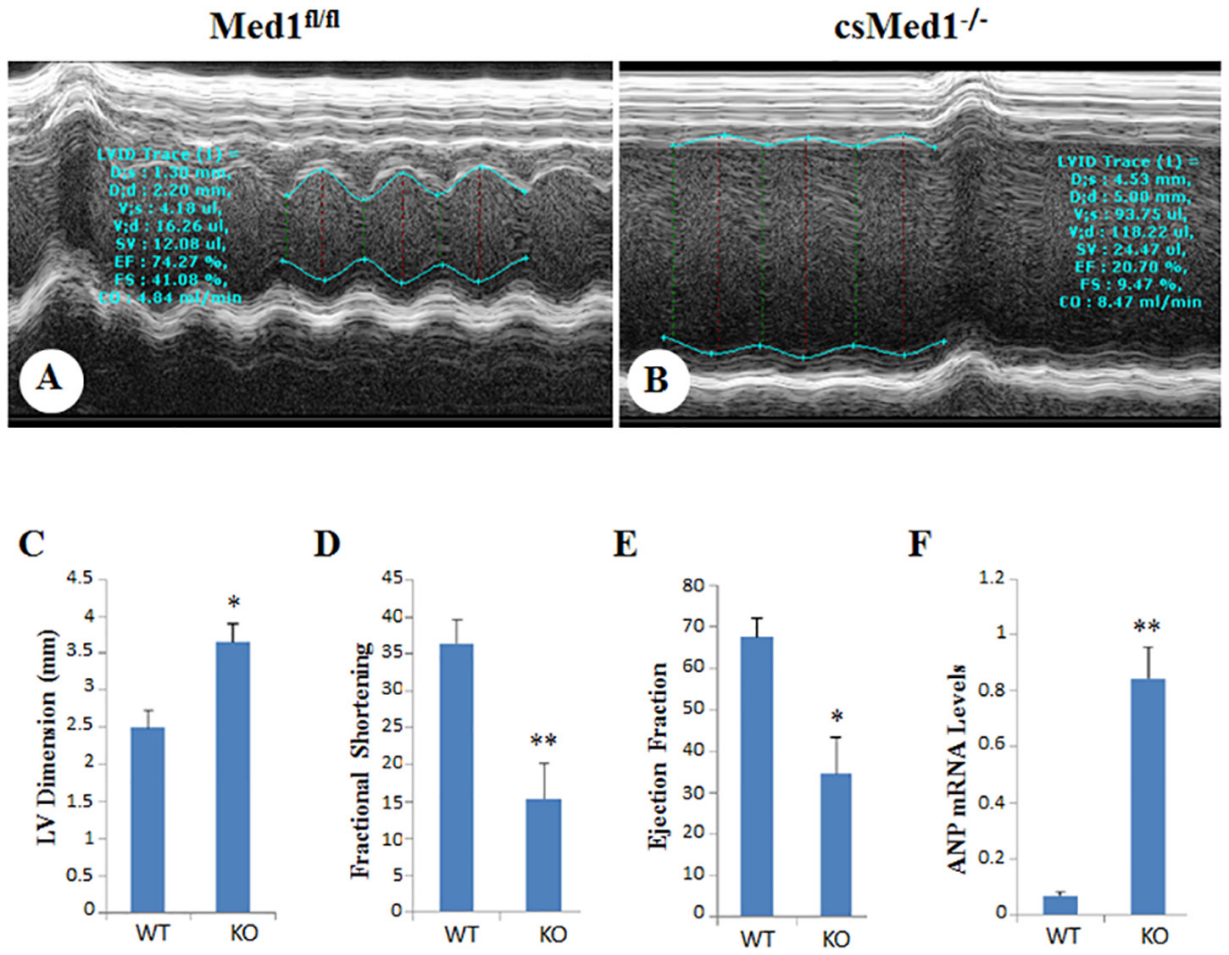
Echocardiography. (A&B) Representative profiles of M-mode echocardiographic analyses of 29-day old Med1^fl/fl^ (A) and csMed1^-/-^ (B) mice. (C, D, & E) Depict left ventricular dimension, fractional shortening and ejection fraction respectively. (F) Relative mRNA levels of ANP (*Nppa*) in Med1^fl/fl^ and csMed1^-/-^ mouse hearts. Results are expressed as the mean ±SD. *p<0.05, **p<0.01.

The effects of Med1 deletion on cardiac function were evaluated by obtaining the 2D and M-mode echocardiographic images (Fig 3A and 3B). Echocardiographic analysis of 23 to 29-day old csMed1^-/-^ mice revealed increased left ventricular end-diastolic internal dimension (LVID-d), decreased fractional shortening and also decreased ejection fraction (Fig 3A, 3B, 3C, 3D and 3E). At day 29, the contractility of csMed1^-/-^ mouse heart was diminished with a fractional shortening of 9.47% vs 41.08% for littermate controls (P<0.001). Likewise, the ejection fraction in 29 day old csMed1^-/-^mouse was 20.70% vs 74.27% for floxed littermate controls. These values suggest poor contractility of Med1 null hearts and support the conclusion that Med1 deficient mice die of heart failure. Accordingly, the mRNA levels of heart failure indicators ANP and BNP were significantly elevated in csMed1^-/-^mouse heart (Figs 1D and 3F). Both ANP and BNP levels increase in heart failure as ventricular cells are recruited to secrete both these peptides in response to left ventricular dysfunction [28].

### Changes in gene expression related to cardiac-specific abnormalities

Because we observed the above described structural and functional abnormalities in the heart of csMed1^-/-^ mice, we considered it important to examine the genes that may be relevant to these abnormalities. First, we carried out expression profile analysis of Med1^-/-^ heart tissue using the RNA-seq approach to obtain a global view of changes in gene expression. Heart RNA samples from 5 controls and 5 csMed1^-/-^ 23 day old mice were pooled and subjected to RNA-seq protocol as described [21]. Using this approach we identified a total of 416 genes with greater than 2-fold expression difference between control and Med1 negative heart RNA samples. Of these, 234 genes showed decreased expression whereas expression of the rest of the genes was elevated.

Gene expression changes in csMed1^-/-^ hearts as analyzed by qPCR as well as RNA-Seq are listed in Tables 1 and 2. The primers used for the real-time PCR are listed in S1 Table. The lists of down- and upregulated genes classified according to KEGG pathway and their role in some of the pathways related to heart function are shown in Tables 3 and 4, respectively. The complete list of down regulated genes is provided in S2 Table. S3 Table lists the classification of down-regulated genes based on biological process, molecular function and cell component. S4 Table shows the list of upregulated genes, and classification of upregulated genes based on biological process, molecular function and cell component is shown in S5 Table. The entire list of genes analyzed by RNA-seq that show significant difference in expression levels has been deposited in Gene Expression Omnibus (GEO accession number is GSE65873). As expected, RNA-seq analysis validated reduced expression of most of the down-regulated genes (Table 1). These genes are involved in calcium signaling, cardiac muscle contraction, cardiac hypertrophy and fibrosis.

**Table 1:** Gene expression changes in csMed1^-/-^ relative to Med1^fl/fl^ mouse heart.

Function	Gene	csMed1^-/-^ /Med1^fl/fl^		csMed1^-/-^ /Med1^fl/fl^
		QPCR	*p* value	RNA-Seq
Energy metabolism				
	*Ppara*	0.087	*	0.131
	*Ppargc1a*	0.067	**	0.215
	*Ppargc1b*	0.035	**	0.091
	*L-PBE*	0.191	*	0.364
	*Acadm*	0.056	**	0.135
	*UCP3*	0.284	*	0.359
	*Abcc9*	0.104	*	0.086
	*Aqp7*	0.079	*	0.137
Glucose metabolism				
	*GK*	0.186	*	0.295
	*HK2*	0.171	**	0.317
	*Glut4*	0.087	*	0.206
Calcium signaling pathway/				
Cardiac muscle contraction				
	*Ryr2*	0.072	*	0.086
	*ATP2a2*	0.049	**	0.066
	*ATP1a2*	0.046	**	0.097
	*Pln*	0.032	**	0.065
	*Ped1c*	0.016	**	0.036
Transcription factor/coactivator				
	*Tfam*	0.125	*	0.256
OXPHOS				
	*Ndufs7*	0.309	*	0.335
	*Cox6a*	0.303	*	NS
	*Cox5b*	0.208	*	NS
	*Sdhb*	0.197	**	0.349
	*Sdhc*	0.187	*	NS
	*Cox10*	0.187	*	0.354
	*Sdhd*	0.155	*	0.301
	*Sdha*	0.125	*	0.291
ROS detoxification				
	*Cat*	0.184	*	NS
	*Sod2*	0.171	**	0.398

*P<0.05

**P<0.01

NS: not significant

**Table 2:** Up-regulated genes in csMed1^-/-^ relative to Med1^fl/fl^ mouse heart.

Function	Gene	csMed1^-/-^ /Med1^fl/fl^		csMed1^-/-^ /Med1^fl/fl^
		QPCR	*p* value	RNA-Seq
Hypertrophic/Dilated/Fibrosis				
Cardiomyopathy/calcium signal Pathway	*Col9a2*	26.69	**	69.83
	*Wisp2*	24.34	**	52.68
	*Inhbb*	9.01	**	19.45
	*Atf3*	7.18	**	12.17
	*Tgfb2*	6.32	**	9.41
	*Ctgf*	4.41	**	12.42
	*Ace*	4.08	**	7.16
	*Mmp3*	3.15	*	6.26
	*Itgav*	2.97	*	6.08
	*Cacnb1*	2.81	*	5.63
	*Cldn15*	2.75	**	8.66
	*Actn1*	2.38	**	8.07
	*Thbs1*	2.38	*	6.97
	*Myl9*	2.15	*	6.11
Metabolism/Apoptosis	*Hspa1b*	16.23	**	22.66
	*Hspa1a*	15.81	**	23.76
	*Cyp1b1*	9.19	**	20.25
	*Cdkn1a*	8.15	**	20.93
	*Angptl4*	5.66	**	9.45
	*Gpx3*	4.67	**	12.05
	*Tlr5*	4.52	*	13.75
	*Map3k6*	3.29	**	5.41
	*Ppp1r5a*	2.34	*	6.56

*P<0.05

**P<0.01

**Table 3:** Genes down-regulated in csMed1^-/-^ heart as assessed by RNA-Seq KEGG pathway.

Gene ID	Gene Name	KEGG_PATHWAY
Acadm	acyl-Coenzyme A dehydrogenase, medium chain	mmu03320:PPAR signaling pathway
Aqp7	aquaporin 7	mmu03320:PPAR signaling pathway
Atp1a2	ATPase, Na+/K+ transporting, alpha 2 polypeptide	mmu04260:cardiac muscle contraction, mmu04972:pancreatic secretion
Atp2a2	ATPase, Ca++ transporting, cardiac muscle, slow twitch 2	mmu04260:cardiac muscle contraction, mmu05412:arrhythmogenic right ventricular cardiomyopathy (ARVC), mmu05414:dilated cardiomyopathy, mmu04020:calcium signaling pathway, mmu04972:pancreatic secretion
Cacna1s	calcium channel, voltage-dependent, L type, alpha 1S subunit	mmu04260:cardiac muscle contraction, mmu05412:arrhythmogenic right ventricular cardiomyopathy (ARVC), mmu05414:dilated cardiomyopathy, mmu04020:calcium signaling pathway
Entpd5	ectonucleoside triphosphate diphosphohydrolase 5	mmu00240:pyrimidine metabolism
Ephb1	Eph receptor B1	mmu04360:ATP binding
Gja1	gap junction protein, alpha 1	mmu05412:arrhythmogenic right ventricular cardiomyopathy (ARVC)
Lpl	lipoprotein lipase	mmu03320:PPAR signaling pathway
Nos2	nitric oxide synthase 2, inducible	mmu04020:calcium signaling pathway
Nt5c1a	5'-nucleotidase, cytosolic IA	mmu00240:pyrimidine metabolism
Pde1c	phosphodiesterase 1C	mmu04020:calcium signaling pathway
Pla2g5	phospholipase A2, group V	mmu04972:Calcium ion binding
Pln	Phospholamban	mmu05414:dilated cardiomyopathy, mmu04020:calcium signaling pathway
Ppara	peroxisome proliferator activated receptor alpha	mmu03320:PPAR signaling pathway
Ryr2	ryanodine receptor 2, cardiac	mmu04260:cardiac muscle contraction, mmu05412:arrhythmogenic right ventricular cardiomyopathy (ARVC), mmu05414:dilated cardiomyopathy, mmu04020:calcium signaling pathway
Scd4	stearoyl-coenzyme A desaturase 4	mmu03320:PPAR signaling pathway

**Table 4:** Genes up-regulated in csMed1^-/-^ heart as assessed by RNA-Seq KEGG pathway.

Gene ID	Gene Name	KEGG_PATHWAY
*Ace*	angiotensin-converting enzyme	mmu05410:Hypertrophic cardiomyopathy, mmu05142:Chagas disease
*Cacnb1*	calcium channel voltage-dependent subunit beta 1	mmu05410:Hypertrophic cardiomyopathy, mmu05412: Arrhythmogenic right ventricular cardiomyopathy (ARVC), mmu05414:Dilated cardiomyopathy, mmu04010:MAPK signaling pathway
*Itgav*	Integrin alpha V	mmu05410:Hypertrophic cardiomyopathy, mmu05412: Arrhythmogenic right ventricular cardiomyopathy (ARVC), mmu05414:Dilated cardiomyopathy, mmu05200:Pathway in cancer, mmu04510:Focal adhesion, mmu04145:Phagosome, mmu04512:ECM-receptor interaction
*Tgfb2*	transforming growth factor, beta 2	mmu05410:Hypertrophic cardiomyopathy, mmu05414:Dilated cardiomyopathy, mmu04010:MAPK signaling pathway, mmu04144:Endocytosis, mmu05200:Pathway in cancer, mmu05145:Toxoplasmosis, mmu05142:Chagas disease, mmu05146:Amoebiasis
*Actn1*	Actinin, alpha 1	mmu05412:Arrhythmogenic right ventricular, cardiomyopathy (ARVC), mmu04530:Tight junction
*Myl9*	myosin, light polypeptide 9, regulatory	mmu04530:Tight junction, mmu04670:Leukocyte transendothelial migration
*Cldn15*	Claudin 15	mmu04530:Tight junction, mmu04670:Leukocyte transendothelial migration
*Hspa1b*	heat shock protein 1B	mmu04010:MAPK signaling pathway, mmu04144:Endocytosis, mmu04612:Antigen processing and presentation
*Map3k6*	mitogen-activated protein kinase kinase 6	mmu04010:MAPK signaling pathway
*Inhbb*	inhibin beta-B	Mmu04350:TGFβ signaling pathway
*Thbs4*	thrombospondin 4	Mmu04350:TGFβ signaling pathway, mmu04145:Phagosome, mmu04510:Focal adhesion, mmu04512:ECM-receptor interaction
*Thbs1*	thrombospondin 1	Mmu04350:TGFβ signaling pathway, mmu04115:p53 signaling pathway, mmu04145:Phagosome

### Down-regulated genes in csMed1^-/-^ heart

#### Fatty acid oxidation abnormalities

**Fig 4:**
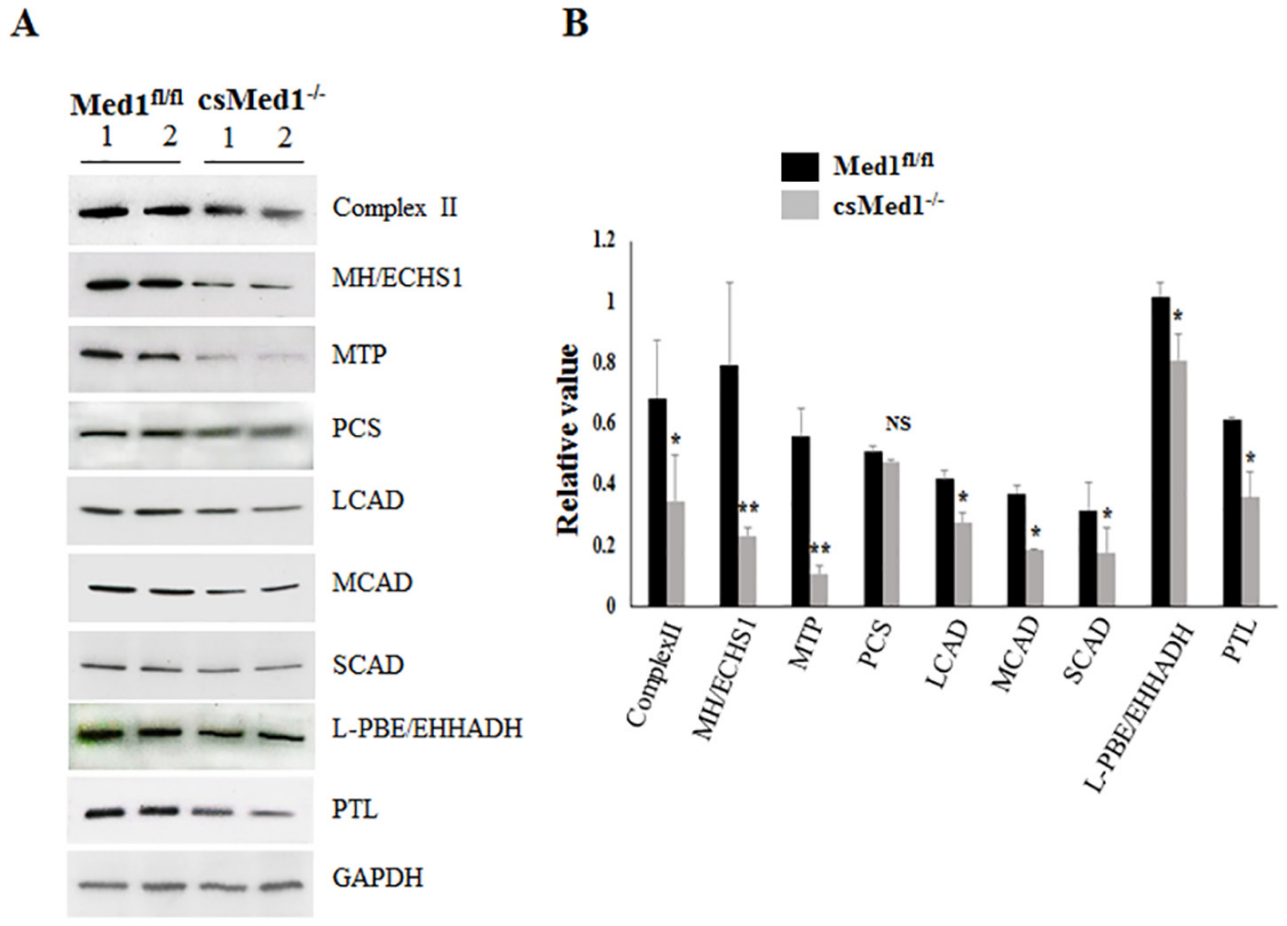
Changes in selected proteins involved in fatty acid oxidation in heart. (A) Homogenates of heart from two 29-day old Med1^fl/fl^ and csMed1^-/-^ mice were immunoblotted with antibodies against the proteins shown. (B) Densitometry of Western blot to analyze the relative protein expression. The protein expression of each gene was normalized to GAPDH. Results are expressed as the mean ±SD. *p<0.05, **p<0.01, NS: non-significant. 2-Enoyl-CoA hydratase (MH); Mitochondrial trifunctional protein (MTP); Pyruvate carboxylase (PCS); 3-ketoacyl-CoA thiolase (PTL); Long chain, medium chain, and short chain acyl-CoA dehydrogenases (LCAD, MCAD, and SCAD, respectively); Enoyl-CoA hydratase/L-3-hydroxyacyl-CoA dehydrogenase (L-PBE).

Med1 is a critical component of the transcriptional regulator Mediator for nuclear receptor *PPARα*, *PPARγ*, *CAR and GR* signaling in liver [19,29–31]. In this regard, conditional deletion of Med1 in hepatocytes has established a role for this coactivator in *PPARα-*regulated transcriptional activation of FAO genes in liver [19]. Since disturbances in *PPARα* and *PPARδ* regulated FAO has been implicated in cardiomyopathy, we inferred that Med1 deletion in cardiomyocytes perturbs myocardial FAO and energy metabolism [32–36] in csMed1^-/-^ hearts (Table 1, Fig 4). qPCR analysis revealed significant reductions in transcripts of *PPARα*, *PPARδ*, *PPARγ*, *Ppargc1α* (PPARγ-coactivator-1α), *Ppargc1β*, *Abcc9*, *Acadm* and others in Med1 null hearts compared with that of floxed littermates (Table 1). Immunoblotting confirmed reductions in the amount of key mitochondrial and peroxisomal fatty acid β-oxidation system proteins such as mitochondrial enoyl-CoA hydratase (ECHS1), mitochondrial trifunctional protein (MTP), medium-chain acyl-CoA dehydrogenase (MCAD), short-chain acyl-CoA dehydrogenase (SCAD), and peroxisomal enoyl-CoA, hydratase/3-hydroxyacyl CoA dehydrogenase (EHHADH/L-PBE) and peroxisomal thiolase (ACAA1/PTL) (Fig 4). These reductions are consistent with the reductions noted in PPARα transcript levels (Table 1).

#### Glucose metabolism

Also evident in our RNA analysis were the down-regulation of *HK2*, *GK*, and *Glut4* genes that are involved in glucose metabolism in csMed1^-/-^ hearts (Table 1). Heart consists of specialized muscle cells (cardiomyocytes), which contract constantly in a coordinated fashion. To maintain its contractile function, heart cells must receive constant supply of metabolic substrates to generate ATP. Although major metabolic substrate for cardiomyocytes is fatty acids, about 30% of the myocardial ATP is generated by glucose and lactose [37]. Glut4 is the most abundant glucose transporter in heart which mediates entry of glucose into heart cells [38]. Thus, reduced expression of Glut4, hexokinase2 and glucokinase would drastically affect heart function.

#### Mitochondrial oxidative phosphorylation (OXPHOS) and calcium homeostasis

Defects in OXPHOS contribute to alteration of mitochondrial membrane potential, reactive oxygen species production and mitochondrial Ca2^+^ homeostasis (Table 1). Accordingly, significant disturbances in the expression levels of key genes, namely *Pde1c*, *Pln*, *Ryr2*, *ATP1a2 and ATP2a2* involved in calcium signaling pathway and cardiac muscle contraction were observed in csMed1^-/-^ hearts (Table 1). Reduced expression of many OXPHOS genes were also observed in csMed1^-/-^ hearts including *Ndufs7*, *Cox10*, *Cox5b*, *Sdha*, *Sdhb*, *Sdhc*, *Sdhd* and others (Table 1).

#### Mitochondrial biogenesis

Our results show a reduced expression of both PGC1α and PGC1β transcriptional coactivators in csMed1^-/-^ heart. Similarly, we also observed an 8-fold reduced expression of TFAM (transcriptional factor A, mitochondrial; Table 1). Both PGC1α and PGC1β were shown to be important in mitochondrial biogenesis [39,40]. Reduced levels of TFAM may also have contributed to mitochondrial biogenesis as expression of TFAM, is involved in mitochondrial DNA transcription and maintenance of mitochondrial genome copy number [40]. Thus reduced expression of these genes could explain the reduced mitochondrial DNA content that we observed in this study (Fig 2).

### Upregulated genes in csMed1^-/-^ heart

The RNA-seq data of csMed1^-/-^ heart shows nearly one half of the differentially expressed genes upregulated (S5 Table). Twenty-three of these genes that are implicated in cardiac dysfunction and hypertrophy were validated by qPCR (Table 2). The genes in this category are linked to a variety of cardiac abnormalities ranging from cardiac signaling pathway, calcium muscle contraction (*Cacnb1*), cardiac hypertrophy and fibrosis and stress response. For example, *TGFβ2*, *Ace*, *Itgav*, and *Cacnb1* were shown to be involved in calcium signaling and cardiac muscle contraction [41–44]. The protein encoded by *Cacnb1* belongs to the calcium channel beta subunit family [45]. It plays an important role in the calcium channel function and also appears to be elevated due to myocardial damage [44]. Expression of angiotensin converting enzyme (ace; Table 3) is also elevated in animal models of heart failure [42]. Induction of both *ItgaV* (integrin alpha v), and *Angptl4* are in response to heart dysfunction and heart failure as these proteins play vital roles in myocardial functions [46,47]. Activating transcription factor 3 (ATF3), a transcription factor of the ATF family is induced by various stress responses [48]. Overexpression of ATF3 in heart results in rapid ventricle hypertrophy and ATF3 KO mice display reduced heart hypertrophy [49,50]. Thus, csMed1^-/-^ mice may have induced ATF3 levels as a protection against cardiac hypertrophy. The gene *Col9a2* is a component of type IX collagen related to cardiac fibrosis and elevated expression of this gene is consistent with fibrosis observed in csMed1^-/-^ mice (Table 2). Similarly, published data suggest that during cardiac hypertrophy heat shock proteins Hspa1a and 1b are elevated [51]. Overall, the 23 upregulated genes that we have validated are involved in one or more aspects of cardiac abnormalities and appear to be in response to the damage occurred in csMed1^-/-^ hearts.

### Confirmation of csMed1 phenotype using tamoxifen inducible heart specific Med1 KO mouse (TmcsMed1^-/-^)

In studies described above, we used mice expressing α-MyHC promoter driven cre recombinase to delete Med1 gene in the heart. As stated earlier, a rapid transition from β- to α-myosin heavy chain expression occurs during rodent development and by postnatal day 21, α-myosin is expressed predominantly [23]. Accordingly, the α-MyHC-Cre is expressed late in the embryonic development and maximally during the postnatal period which contributes to Med1 deletion resulting in the death of csMed1^-/-^ mice within ten days after weaning [23]. To firmly establish that the lack of Med1 expression is solely responsible for all the heart abnormalities and the associated heart failure observed in csMed1^-/-^ mice, we used tamoxifen-inducible heart-specific Cre (Myh6-MCM)/Med1^fl/fl^ mouse model (TmcsMed1^-/-^). The tamoxifen-inducible gene knockout strategy has clear advantages in that expression of a gene can be ablated in adult mice at will in a tissue specific manner [20].

**Fig 5:**
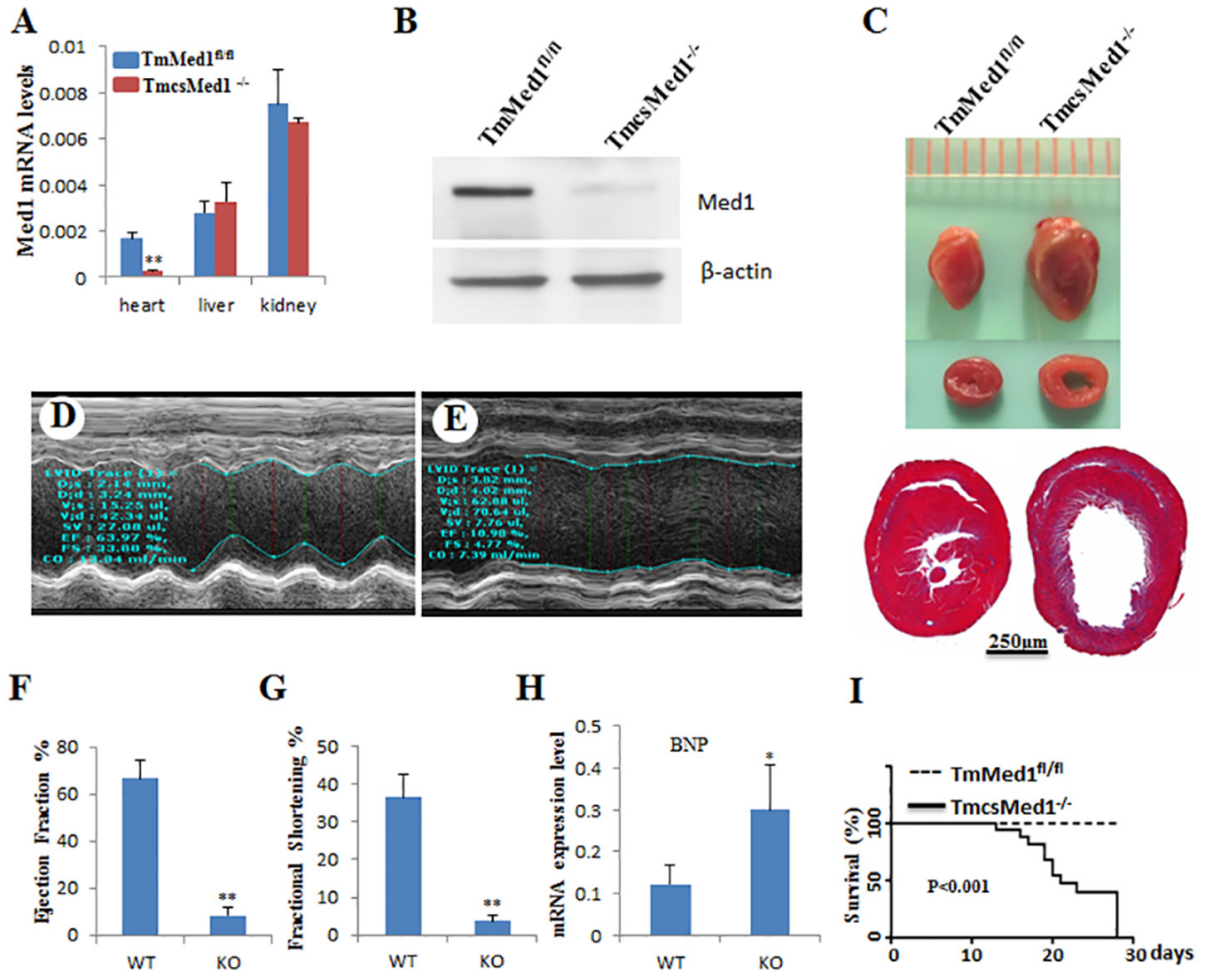
Tamoxifen-inducible cardiac-specific disruption of Med1 (TmcsMed1^-/-^) in adult mice causes dilated cardiomyopathy. (A) Relative Med1 mRNA expression in Med1^fl/fl^ and TmcsMed1^-/-^ mouse heart, liver and kidney. (B) Western blot analysis of Med1 in TmMed1^fl/fl^ and TmcsMed1^-/-^ hearts. Crude nuclear extracts from the heart tissues of appropriate mice were prepared as described (see Materials and Methods). They were then Western immunoblotted and probed with an anti-Med1 antibody (Abcam ab64965) and the protein bands were quantified using ImageJ software. The data were normalized to β-actin bands. Note that in the experiments shown in (A) to (H), mice were killed 14 days after first tamoxifen injection. (C) Representative photographs of adult hearts after tamoxifen-inducible heart-specific Cre mediated Med1 deletion. It is evident that TmcsMed^-/-^ mouse heart is flaccid and flabby. Lower panel in (C) shows cross sections of TmcsMed1^-/-^ and the littermate control hearts stained with H&E. (D and E) Representative profiles of M-mode echocardiographic analyses of TmcsMed1^-/-^ and littermate mice. (F) and (G) represent ejection fraction and fractional shortening respectively. Data were derived from (D) and (E). (H), relative mRNA levels of BNP (*Nppb*) in Med1 ^fl/fl^ and TmcsMed1^-/-^ mouse hearts. (I), survival curve. 13 mice for each group of Med1^fl/fl^ and TmcsMed1^-/-^ were used for the generation of survival curve. The mice survival time was between 13 and 28 days, and the day of initial injection of Tamoxifen was counted as day 1. Results are expressed as the mean ±SD. *p<0.05, **p<0.01.

To study the role of Med1 in adult heart, 7-week old TmcsMed1^-/-^ mice were given a daily Iintraperitoneal injection of tamoxifen at a dose of 65mg/kg for 5 days and killed at selected intervals thereafter. qPCR analysis of RNA showed that the Med1 expression began to decrease after 3 days of tamoxifen injection (about 70% decrease; data not shown), and by 5 days of injection, Med1 expression was almost non-detectable in the heart (Fig 5A). The Med1 expression levels in liver and kidney of the same mice did not change when compared to control mice (RNA data in Fig 5A). Med1 expression in TmcsMed1^-/-^ was also evaluated using Western blot method. Crude nuclear extracts were prepared from csMed1^fl/fl^ and TmcsMed1^-/-^ hearts then subjected to Western immunoblots. Fig 5B shows that the Med1 expression was negligible in TmcsMed1^-/-^ hearts as compared to that of TmMed1^fl/fl^ hearts. Quantification of the Med1 protein bands shown in this figure indicated that Med1 levels decreased by about 90% when compared to that of littermate controls (see Materials and Methods for Western blot and protein band quantification details). The residual Med1 observed here may due to non cardiomyocte nuclei. including from fibroblasts. Additionally, immunostaining of the relevant heart tissues confirmed this observation (S1 Fig). The size of TmcsMed1^-/-^ heart increased dramatically as compared to littermate controls and the Med1 deficient hearts appeared increasingly flaccid by 14 days after the first tamoxifen injection (Fig 5C, *upper panel*). As shown for the csMed1^-/-^, the histological examination of TmcsMed1^-/-^ mouse hearts also revealed dilatation of the right and left ventricular chambers with thinning of the walls and myocardial cells (Fig 5C, lower panel).

Echocardiographic analysis TmcsMed1^-/-^ mice revealed increased left ventricular end-diastolic internal dimension (LVID-d), decreased fractional shortening and also decreased ejection fraction (Fig 5D and 5E). The contractility of TmcsMed1^-/-^ mouse heart was diminished with the ejection fraction in TmcsMed1^-/-^ mouse was only 11% vs 64% for floxed littermate controls (Fig 5F; P<0.001. Likewise, a fractional shortening of 4.77% vs 33.88% for littermate controls (Fig 5G; P<0.001) was also observed. These values suggest poor contractility of Med1 null hearts in TmcsMed1^-/-^ mice and support the conclusion that Med1 deficient mice die of heart failure. Accordingly, the mRNA levels of heart failure indicator BNP was significantly elevated in TmcsMed1^-/-^ mouse heart (Fig 5H). The survival period of TmcsMed1^-/-^ mice was between 13 and 28 days, and the day of initial injection of Tamoxifen was counted as day 1 (Fig 5I).

**Fig 6:**
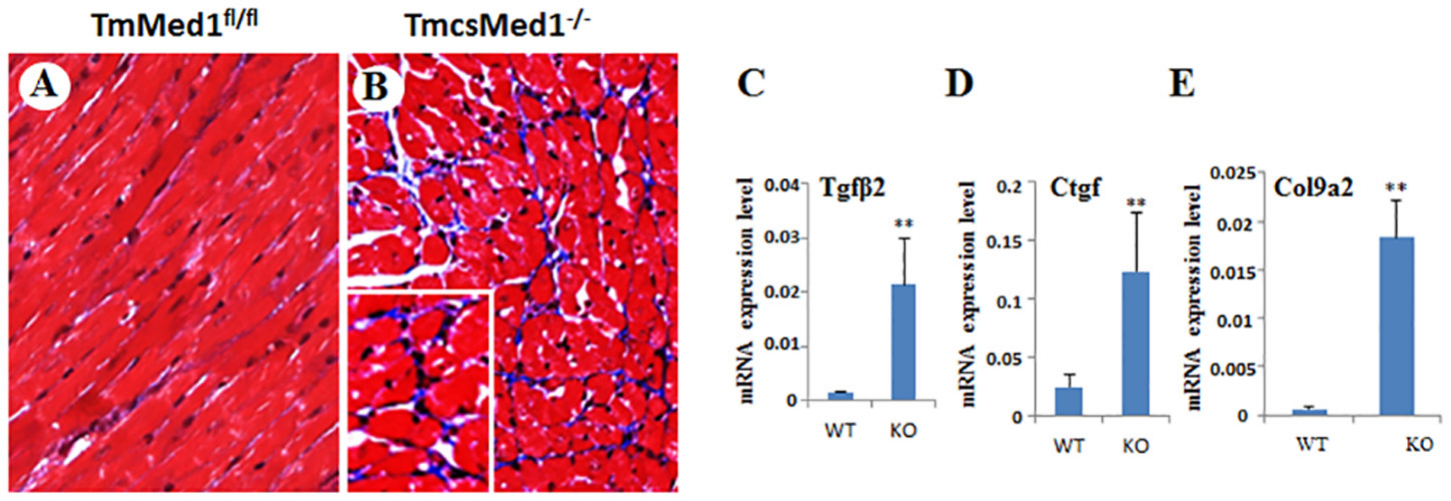
Cardiac fibrosis in TmcsMed1^-/-^ mouse heart. TmcsMed1^-/-^ mice given daily injections of tamoxifen for 5 days were killed 14 days after the first tamoxifen injection. (A&B) Masson’s trichrome staining was used to assess myocardial fibrosis. *Inset* in panel B shows intensely stained (*blue color*) interstitial fibrous strands. (C,D &E) Quantification of mRNA levels for *Tgfβ2*, *Ctgf and Col9a2* mRNA levels in TmcsMed1^-/-^ (KO) and TmMed1^fl/fl^ (WT) mouse hearts. mRNA levels were quantified by qPCR assays and the results were expressed as the mean ±SD. **p<0.01.

We also examined the extent of apoptosis of cardiomyocytes in TmcsMed1^-/-^ heart. The formalin fixed paraffin-embedded heart sections by TUNEL staining showed evidence of enhanced apoptosis in TmcsMed1^-/-^ mouse hearts compared with that noted in normal hearts (S2A Fig). In the TmcsMed1^-/-^ mouse heart the number of apoptotic cardiomyocytes increased by about 10-fold (S2B Fig). Masson trichrome staining showed evidence of interstitial myocardial fibrosis in TmcsMed1^-/-^ heart (Fig 6A and 6B). Previous studies have shown that interstitial myocardial fibrosis is associated with increased expression of TGFβ2, CTGF and collagenase 9a2 [52,53]. In agreement with this, the TmcsMed1^-/-^ heart showed elevated expression of the above three genes (Fig 6C, 6D and 6E). This result is highly comparable to that we observed in csMed1^-/-^ heart (see Fig 2A). However, it appears that fibrosis is more pronounced in TmcsMed1^-/-^ heart when compared to that of csMed1^-/-^ heart (see MT panels in Fig 2A).

**Fig 7:**
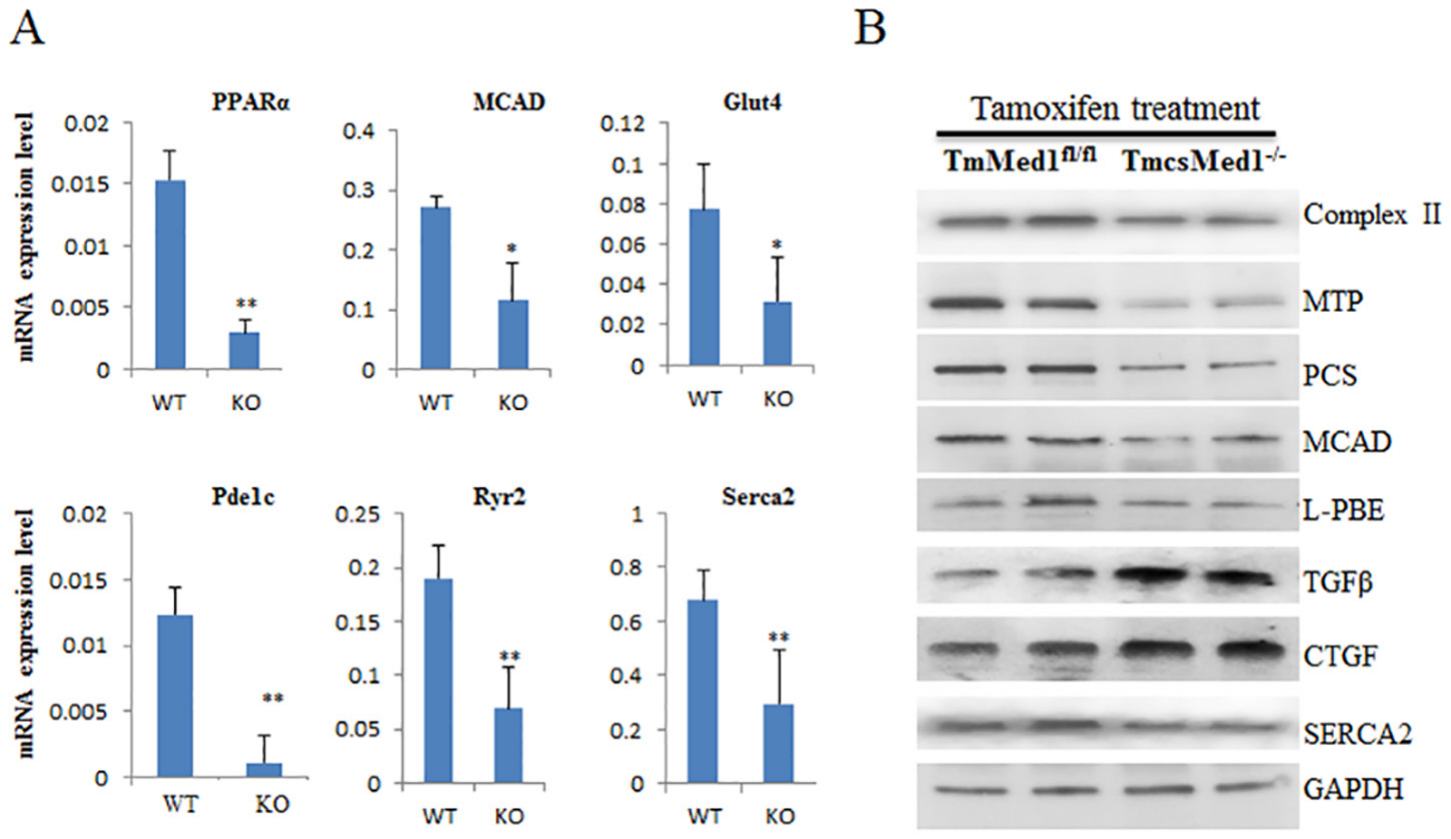
Analysis of expression of selected genes in Med1^fl/fl^ and TmcsMed1^-/-^ mouse heart. (**A**) qPCR data of RNA samples for key genes linked to heart function. (**B**) Western blot analysis of key proteins. Protein homogenates extracted from hearts of tamoxifen-inducible Med1^fl/fl^ (14 days after initial injection) and TmcsMed1^-/-^ mice were immunoblotted. The protein bands were quantified using a densitometer. Percent change for TmcsMed1^-/-^ heart as compared to littermate controls are: MTP (Complex II), 28% reduction; MTP (Mitochondrial trifunctional protein), 67% reduction; PCS (Pyruvate carboxylase), 61% reduction; MCAD (Medium chain acyl-CoA dehydrogenases), 42% reduction; L-PBE (Enoyl-CoA hydratase/L- hydroxyacyl-CoA dehydrogenase), 44% reduction; Tgfβ (transforming growth factor beta), 119% increase; CTGF (connective tissue growth factor), 68% increase; SERCA2 (ATPase, Ca++ transporting, cardiac muscle, slow twitch 2), 29% reduction). The protein expression was normalized to GAPDH.

In studies using csMed1^-/-^ model, our RNA-seq analysis showed down-regulation of a large number of genes in heart that impinge on several pathways including energy and calcium channeling pathways (Table 3). We confirmed these observations in TmcsMed1^-/-^ mice using a set of key genes related to these two pathways using qPCR assays. For example, PPARα, MCAD and Glut4 genes which are involved in energy pathways are reduced significantly in TmcsMed1^-/-^ mice (Fig 7A). Similarly Pde1c, Ryr2 and Serca2 which are related to calcium channeling are also decreased several fold (Fig 7A). Expression of Tgfβ2, and Ctgf genes involved in cardiac fibrosis was upregulated in TmcsMed1^-/-^ mouse heart. Thus, the gene expression changes observed in both csMed1^-/-^ and TmcsMed1^-/-^ hearts are highly comparable.

Expression of several genes involved in mitochondrial function and fatty acid β-oxidation (Complex II, CPT, PCS, MCAD and L-PBE), and calcium channeling (SERCA2) were evaluated using Western blots. As shown in the Fig 7B, protein levels for these genes were decreased. These results are quite similar to those we observed in csMed1^-/-^ hearts (note that Serca2 was analyzed by RNA-seq analysis in csMed1^-/-^ hearts). Protein levels of cardiac fibrosis related genes Tgfβ and CTGF were increased in TmcsMed1^-/-^ heart consistent with the development of cardiac fibrosis in TmcsMed1^-/-^ mice (Fig 7B: see legend to Fig 7B for quantification data). Overall, it is clear that the heart abnormalities and the gene expression changes that we observed in csMed1^-/-^ mice are highly comparable to the findings noted in TmcsMed1^-/-^ heart.

## Discussion

The mechanisms involved in the control of tissue specific gene expression are complex in which a number of transcription factors, coactivators, and epigenetic factors coordinate at the promoter of each gene to initiate transcription. Central to this mechanism is the Mediator complex consisting of about 30 subunits that integrates diverse transcriptional signals and instructs RNA polymerase II and associated proteins to initiate gene specific transcription [1,3]. A detailed understanding of how each of these proteins coordinate with each other and with the Mediator complex has the potential to develop drugs or other interventional strategies for the treatment of diseases arising in different organs.

In this study, we have for the first time investigated the role played by the major subunit of the Mediator complex Med1 in the myocardial function. Mice with Cre-mediated deletion of Med1 in myocardium (csMed1^-/-^) die within a few days after weaning due to heart failure. Anatomical, histological and gene expression studies indicate that mice die of dilated cardiomyopathy with both right and left enlarged ventricles. Our results also indicate considerable disruption of sarcomere structure and abnormalities of mitochondrial ultrastructure and decreased mitochondrial biogenesis. All of these observations are consistent with the changes in gene expression patterns as revealed by qPCR assays and RNA-seq analysis.

As stated above, csMed1^-/-^ mice were generated using α-MyHC-Cre transgenic mice to delete Med1 gene. In this model, the α-MyHC-Cre expression occurs late in the embryonic development and maximally during the early postnatal period [23]. Med1 deletion by this approach results in death of mice within ten days of weaning. Subsequently, we used tamoxifen-inducible Cre approach to delete Med1 gene in adult heart [12]. Analysis of the TmcsMed1^-/-^ heart showed that all of the observations made in csMed1^-/-^ heart were also true for the TmcsMed1^-/-^ heart including enlargement of heart, flaccid nature of the heart tissue, apoptotic cell death of myocytes, and interstitial myocardial fibrosis. Changes in gene expression that occurred in csMed1^-/-^ hearts were also observed in TmcsMed1^-/-^ heart. In summary, we confirmed the essential phenotypic abnormalities noted in csMed1^-/-^ mouse heart also in the Med1 deleted adult heart using the tamoxifen- inducible-Cre approach.

Heart derives most of its energy from fatty acid oxidation [33]. Gene expression analysis of csMed1^-/-^ heart showed that expression of PPARα, a pivotal regulator of energy homeostasis is decreased by about 11-fold. A moderate decrease in PPARγ and PPARβ/δ was also observed (2 to 3-fold). The expression of the two key coactivators of the PPAR subfamily of nuclear receptors, namely PGC1α and PGC1β is also decreased dramatically in csMed1^-/-^ heart tissue (13- and 25-fold respectively). Reduced expression of these genes in csMed1^-/-^ myocardium is consistent with the previously published results demonstrating that Med1 plays a critical role in the transcriptional activation of genes regulated by nuclear receptors such as PPARα [19,29,54]. In the heart, almost every step in the utilization of fatty acids including uptake of fatty acids, their esterification, and every enzyme in the β-oxidation of fatty acids is controlled by PPARα [55,56]. Therefore, rapid decline in heart function as a consequence of reduced expression PPARα that occurs following ablation of Med1 is not unexpected. Also noteworthy is that expression of some of key enzymes involved in glucose metabolism including HK2, GK and Glut4 genes is reduced in csMed1^-/-^ hearts. As stated earlier, to maintain its contractile function, heart cells must receive constant supply of metabolic substrates to generate ATP. Although fatty acids are considered as major metabolic substrate for ATP generation by cardiomyocytes, about 30% of the myocardial ATP is generated by glucose and lactose [37]. Therefore, reduced expression of Glut4, HK2 and GK would drastically affect this back up energy source and affect heart function. Thus, when Med1 is deleted in myocardial cells, the entire energy source appears to be blocked.

Dramatically decreased levels of *Pgc1α* and *Pgc1β* gene expression in csMed1^-/-^ myocardium would impact on the expression of a cascade of genes involved in mitochondrial biogenesis and energy homeostasis [39,57,58]. Gene knockout studies have shown that both PGC1α and PGC1β control a subset of overlapping target genes involved in mitochondrial biogenesis and oxidative phosphorylation. In developing heart there is a rapid increase in PGC1α expression before a large burst of mitochondrial biogenesis [39]. Overexpression of PGC1α in transgenic mice in a cardio-specific manner or PGC1β in mammalian cells in culture led to uncontrolled mitochondrial proliferation. These results suggest that optimum amounts of PGC1α and PGC1β are critical for the mitochondrial biogenesis. Therefore, our observation that decreased mitochondrial DNA content in the heart tissue (Fig 2) vis a vis decreased number of mitochondria in csMed1^-/-^ mice suggests that Med1 might be an upstream activator in the gene expression cascade involved in mitochondrial biogenesis. We showed that the expression of mitochondrial transcription factor TFAM is reduced 8-fold. Reduced levels of TFAM may also contribute to mitochondrial biogenesis as expression of TFAM, a multifunctional transcription factor, is involved in mitochondrial DNA transcription and maintenance of mitochondrial genome copy number [40].

The only reported cases of Med1 association with human disease are the overexpression of Med1 in tissues of breast [59] and prostate cancers [60]. A large number of inherited mutations were shown to contribute to different forms of cardiomyopathy including DCM, ARVC, restrictive and hypertrophic cardiomyopathy [17]. However, these mutations do not map to any of the known members of the Mediator subunits. Some of the human Mediator subunit mutations that are associated with cardiovascular diseases (not directly associated with cardiomyopathy) include Med13 that affects hypoxia [61], Med13L which displays transposition of the great arteries and mental retardation [62], and Med15 that causes conotruncal heart malformations [63]. Also of interest is that analysis of a liver-specific Med23-knockout mouse, displayed improved glucose and lipid metabolism [64], suggesting that some subunits of Mediator may have opposing metabolic actions. To our knowledge genetic mapping of the mutations of Mediator subunits in human hearts has not been carried out so far. Our studies provide an incentive to map the Med1 mutations in human hearts in clinical settings. The phenotype that we observed in csMed1^-/-^ mouse heart mirrors in many respects to that observed for mice that carry a zeitgeist recessive N-ethyl-N-nitrosourea (ENU)-induced missense mutation in Med30 gene [32], including DCM, myocardial fibrosis, echocardiographic results, and changes in gene expression patterns. Genes that control metabolic pathways related to oxidative phosphorylation and energy metabolism decline in both these mice. These similarities are striking and intriguing which raises the possibility that some of the transcription factors and coactivators that regulate heart functions may need to interact with both Med1 and Med30 subunits (one subunit does not compensate for the other). Future work will address these possibilities.

We showed that several genes critical for calcium channel structure and function and cardiac muscle contraction are down-regulated significantly in csMed1^-/-^ heart. Ryanodine receptor 2 gene (*Ryr2)* that encodes ryanodine receptor 2 protein triggers a cardiac contraction by mediating Ca2^+^ channeling [65]. *Ryr2* controls mitochondrial Ca2^+^ and ATP levels and a cascade of transcription factors controlling metabolism and survival [66]. Atp2a2 encodes Ca2β ATPase isoform 2a protein (also known as SERCA-2a) and Cacna1s encodes a structural protein of voltage gated calcium channel. SERCA-2a and phospholamban (product of *pln*) in conjunction with integrin linked kinase (ILK) regulates calcium mediated changes in cardiomyocyte contractility [67,68]. *Cacna1S* gene encodes a protein, which is a member of the 10 subunit structure of voltage-gated calcium channel that mediates calcium entry into cardiac and skeletal muscle cells and activates receptor signaling. Cardiac excitability is primarily dependent on proper calcium transport in myocardial cells. Therefore, deregulation of any of these genes such as *Atp2a2* can affect calcium signaling pathway and cardiac muscle contraction which may contribute to DCM and ARVC [69]. A number of clinical conditions related to cardiovascular disease including DCM are caused by genetic mutations in genes (e.g. *RYR2*) that control calcium release from the sarcoplasmic reticulum [35,69,70].

Given that PPARα is down-regulated 11-fold, and that Med1 is essential for PPARα signaling, we wondered whether genes involved in the calcium channeling whose expression levels are also reduced in the heart of csMed1^-/-^ mice are responsive to PPARα. We searched for the PPRE (proliferator-activated receptor (PPAR) response element) using MatInspector program from Genomatix (http://www.genomatix.de/) and identified one putative PPRE with very high matrix score (0.902) on 1.5 kb upstream sequence of *Cacna1S* gene. Putative PPREs with significant matrix score were also found on the 5 kb upstream sequence of *Ryr2* (9 motifs) and *Atp2a2* (8 motifs). All identified PPREs contain direct repeats of hexanucleotide spaced by a single nucleotide (DR-1) [71]. Based on this bioinformatics data, we presume that some of these PPREs are functional which may recruit PPARα (also PPARγ and PPARδ) on to the calcium channeling gene promoter for their transcriptional regulation, supporting the notion that Med1 stabilizes PPARα bound cofactors. Thus our study raises the possibility that calcium signaling members as a novel target for PPARs.

We have observed elevated expression of a large number of genes in csMed1^-/-^ heart. Genes in this category included those that are involved in calcium signaling pathway (cacnb1), stress response pathway, cardiac hypertrophy and fibrosis (see above, Results). Most likely, the increased expression of these genes is due to cardiac damage that occurs due to decreased expression of genes related to cardiac functions. Alternatively, elevated expression of some of these genes may also have contributed to the heart damage. We believe that expression of these genes is normally repressed in heart by a mechanism involving Med1 and increased expression of at least some of these genes due to Med1 deletion may be to mitigate the injury caused by the abrogation of Med1 functions in myocardium.

The molecular mechanisms by which the Med1 deletion results in the up- or downregulation of cardiac gene expression may be complex. Since the Mediator complex is involved in the transcriptional activation mechanism by a variety of ways including by interacting with sequence specific transcription factors, different coactivators such as histone acetylases and also several components of the polII initiation complex [2], it is at present difficult to pinpoint a specific protein-protein interaction causing Med1 related downregulation of cardiac gene expression. Similar explanation may also be offered for the induction of several genes in TmcsMed1^-/-^ mice observed in this study. However, an elegant recent study showed that Med13 transcriptionally represses the nuclear receptor NURR1 and MEF2 transcription factors in skeletal muscle and thereby suppresses the genes involved in glucose uptake and metabolism [72]. Therefore, further studies will unravel the mechanism by which Med1 deletion causes perturbations of cardiac gene expression in TmcsMed1^-/-^ mice.

An intriguing observation is that even though Med1 is the major component of the Mediator complex whose function is vital for the nuclear receptor mediated transcription, deletion of Med1 did not shut down global transcription. Currently, it is not clear whether a functional mediator complex can form in vivo in the absence of Med1 subunit and whether such complex can display residual transcriptional activity. In this regard, we earlier showed that mice with liver specific deletion of Med1 survive for several months [73,74] and liver transcription in this mouse is not globally shut down [74].

In conclusion, we have shown that Med1 subunit of the Mediator complex plays a critical role in myocardial function and occupies a central role connecting multiple pathways involved in heart function. Med1 subunit of the mediator complex is primarily known for its interaction with a number of nuclear receptors. Many of the changes we observed in the heart of csMed1^-/-^ can be explained by the lack of these interactions. Clearly, further studies such as knock-in and in vitro studies are needed to unravel molecular basis of pathological observations we made in the heart tissue of csMed1^-/-^ mice. This initial work will pave the way for those future studies.

## Supporting Information

S1 Fig Immunohistochemical localization of Med1 (shown by an arrow) in csMed1^fl/fl^ and TmcsMed1^-/-^hearts. (TIF)

S2 Fig Cardiomyocyte apoptosis in TmcsMed1^-/-^ mouse heart by TUNEL staining (A), and quantification of TUNEL positive cells (B). (TIF)

S1 Table Primer sequences used for qPCR assays. (DOCX)

S2 Table Genes down-regulated in csMed1^-/-^ heart. (XLSX)

S3 Table Classification of downregulated genes (RNA-Seq data) based on biological process, molecular function and cell component. (DOCX)

S4 Table Genes up-regulated in csMed1^-/-^ heart. (XLSX)

S5 Table Classification of upregulated genes (RNA-Seq data) based on biological process and functions and cell component. (DOCX)
