# Correction: Cardiomyocyte-Specific Ablation of Med1 Subunit of the Mediator Complex Causes Lethal Dilated Cardiomyopathy in Mice

**DOI:** 10.1371/journal.pone.0164316

**Published:** 2016-09-30

**Authors:** Yuzhi Jia, Hsiang-Chun Chang, Matthew J. Schipma, Jing Liu, Varsha Shete, Ning Liu, Tatsuya Sato, Edward B. Thorp, Philip M. Barger, Yi-Jun Zhu, Navin Viswakarma, Yashpal S. Kanwar, Hossein Ardehali, Bayar Thimmapaya, Janardan K. Reddy

Information is missing from the Funding section. Yashpal S. Kanwar was also a recipient of National Institutes of Health, National Institute of Diabetes and Digestive and Kidney Diseases (US)(NIHNIDDK)RO1DK60635. The correcting Funding Disclosure Statement should read as follows: This work was supported by the following sources of funding: National Institutes of Health, National Institute of Diabetes and Digestive and Kidney Diseases(US) (NIHNIDDK)RO1DK097240, Recipient: Janardan K. Reddy; National Institutes of Health, National Institute of Diabetes and Digestive and Kidney Diseases (US) (NIHNIDDK)RO1 DK083163, Recipient: Janardan K. Reddy; National Institutes of Health, National Institute of Diabetes and Digestive and Kidney Diseases (US)(NIHNIDDK)RO1DK60635, Recipient: Yashpal S Kanwar; and National Institutes of Health (US), National Institute of Allergy and Infectious Diseases(NIH) (AI)R21AI1094296, Recipient: Bayar Thimmapaya.
