# Supplementary figures and images for: Cardiomyocyte-Specific Ablation of Med1 Subunit of the Mediator Complex Causes Lethal Dilated Cardiomyopathy in Mice
Source: PLoS One. 2016 Aug 22;11(8):e0160755. doi: 10.1371/journal.pone.0160755 (PMC4993490; doi:10.1371/journal.pone.0160755)

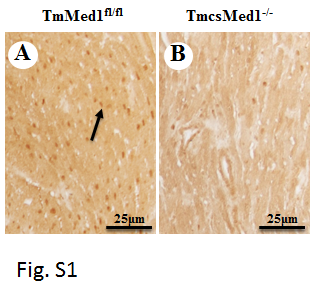

Supplement: S1 Fig — (TIF) [file pone.0160755.s001.tif]

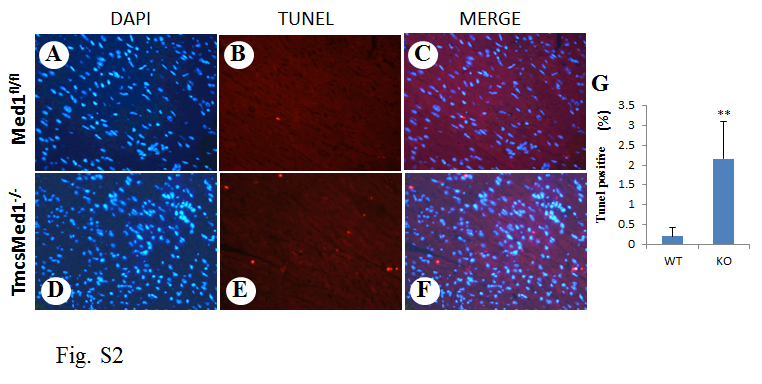

Supplement: S2 Fig — (TIF) [file pone.0160755.s002.tif]
