# Supplementary material for: Cardiomyocyte-Specific Ablation of Med1 Subunit of the Mediator Complex Causes Lethal Dilated Cardiomyopathy in Mice
Source: PLoS One. 2016 Aug 22;11(8):e0160755. doi: 10.1371/journal.pone.0160755 (PMC4993490; doi:10.1371/journal.pone.0160755)
Supplement: S1 Table — (DOCX) [file pone.0160755.s003.docx]

**S1 Table. Selected primers used for qPCR**

| ***Gene name*** | ***Forward primers*** | ***Reverse primers*** |
| --- | --- | --- |
| ***PPARα*** | ***GGGCTCCGAGGGCTCTGTCA*** | ***TGCAGCTCCGATCACACTTGTCG*** |
| ***PGC1α*** | ***CTCCATGCCTGACGGCACCC*** | ***GCAGGGACGTCTTTGTGGCT*** |
| ***PGC1β*** | ***CCGAGCTCTTCCAGATTGAC*** | ***TTCATCCAGTTCTGGGAAGG*** |
| ***L-PBE/EHHADH*** | ***GGTCGTTGGAGTTCCTGTTGCT*** | ***TGGGCAAGCTTGGGACTGGC*** |
| ***UPC3*** | ***GGATGTGGTAAAGACCCGAT*** | ***AGGGCACAAATCCTTTGTAGA*** |
| ***Abcc9*** | ***GGATAGTCCAGCGAGTAAATG*** | ***AGAAAGGTCCTCTGTAGGATAA*** |
| ***Acadm(MCAD)*** | ***GGCCAGAAGATGTGGATAAC*** | ***GTCGGCTTCCACAATGAA*** |
| ***Aqp7*** | ***GCCACCTATCTTCCTGAATAC*** | ***CGGTAACAAGGATGCCTATC*** |
| ***HK2*** | ***GAACCAGATCTACGCCATTC*** | ***GGGAACGAGAAGGTGAAAC*** |
| ***Glut4*** | ***TCTCCTGCAGCTGATTCT*** | ***CTCTCTCCAACTTCCGTTTC*** |
| ***Pde1c*** | ***TAGACAGGGTGACAGAGAAG*** | ***CTGGGAGCTTTCGTCAATTA*** |
| ***Pln*** | ***AAAGTGCAATACCTCACTCG*** | ***GATCAGCAGCAGACATATCAA*** |
| ***Ryr2*** | ***GATCAGCAGACTGGAAAGAC*** | ***GGCATACACAGAGAGAACAC*** |
| ***Atp1a2*** | ***GGGTGGCAAGAAGAAACA*** | ***ATTGGTGAGGCCCTTAGA*** |
| ***Atp2a2(Serca2)*** | ***GTGTATCGACAGGACAGAAAG*** | ***CTTGATGACGGAGACAGATTC*** |
| ***Med1*** | ***GTGTGGTAATGGATGTGCAGGAC*** | ***AGCTTTCCTCCGAATAGCCCTC*** |
| ***ATF3*** | ***TGACACCCTTTGTCAAGGAA*** | ***GCCTCAGACTTGGTGACTGA*** |
| ***Tfam*** | ***CAAGTCAGCTGATGGGTATG*** | ***TGAGCCGAATCATCCTTTG*** |
| ***Ndufs7*** | ***CCTGTTTGTCACAGGACGAC*** | ***AATAGACACCAGCGGAAACC*** |
| ***Sdha*** | ***CTACAAGGGACAGGTGCTGA*** | ***GAGAGAATTTGCTCCAAGCC*** |
| ***Sdhb*** | ***GAATGCAGACGTACGAGGTG*** | ***AGAGCCACAGATGCCTTCTC*** |
| ***Sdhc*** | ***TCAGAAATGCTGCTCCTTTG*** | ***GGGAGACAGAGGACGGTTT*** |
| ***Sdhd*** | ***CTCGAGCTCTCCTACTCCGA*** | ***ACAGGTGAATGTGCTGGGTA*** |
| ***COX5b*** | ***GACCGGGCGTTGTTAGAC*** | ***AACAGGCTGAGCAAGATGG*** |
| ***COX6a*** | ***GAGTTCATCCCGTATCACCA*** | ***CGTGTCTGCTGAGACATCAA*** |
| ***COX10*** | ***TTCCTCAAGCGCATGTATGT*** | ***CTCTAGCCTGCCCTGAAGAC*** |
| ***Sod2*** | ***CAACTCAGGTCGCTCTTCAG*** | ***CAGCAACTCTCCTTTGGGTT*** |
| ***Catalase*** | ***GTCTTCGTCCCGAGTCTCTC*** | ***CTGCCTCTCCATCTGCATTA*** |
| ***ANF(Nppa)*** | ***TACAGTGCGGTGTCCAACA*** | ***CTTCTACCGGCATCTTCTCC*** |
| ***BNP(Nppb)*** | ***GAGTCCTTCGGTCTCAAGGC*** | ***ACAACTTCAGTGCGTTACAGC*** |
| ***Collagen1*** | ***TCCCCGAGGAAACAATGGTG*** | ***CCAATGGGACCAGTCAGACC*** |
| ***18S*** | ***AAACGGCTACCACATCCAAG*** | ***CCTCCAATGGATCCTCGTTA*** |
| ***Itgav*** | ***TGCTGATGGCCAGGGATTTT*** | ***TGATGCTGTAGACATTTGGGTCA*** |
| ***Inhbb*** | ***GTCCGCCTGTACTTCTTCGT*** | ***ACACCTTGACCCGTACCTTC*** |
| ***Ctgf*** | ***CTTCCCGAGAAGGGTCAAG*** | ***CAGTCGGTAGGCAGCTAGG*** |
| ***Ace*** | ***GCCTGGGACTTCTACAACCG*** | ***CTCATGGAAGCCAGGGTTGG*** |
| ***Hspa1b*** | ***CAAGAAGAAGGTGCTGGACA*** | ***GGTACAGCCCACTGATGATG*** |
| ***Hspa1a*** | ***CAAGAAGAAGGTGCTGGACA*** | ***GGTACAGCCCACTGATGATG*** |
| ***Cyp1b1*** | ***CAGCCAGGACACCCTTTC*** | ***CCGGGTATCTGGTAAAGAGG*** |
| ***Wisp2*** | ***AATACAGGTGCCAGGAAGGT*** | ***AGAAAGTTGGTGTCCTTGGG*** |
| ***Tgf β2*** | ***GCGCTACATCGATAGCAAGG*** | ***TAGACGGCACGAAGGTACAG*** |
| ***Col9a2*** | ***TTGTGTCCAACCAACTGTCC*** | ***CTGGATGACCCTTCACTCCT*** |
