# Supplementary material for: Cardiomyocyte-Specific Ablation of Med1 Subunit of the Mediator Complex Causes Lethal Dilated Cardiomyopathy in Mice
Source: PLoS One. 2016 Aug 22;11(8):e0160755. doi: 10.1371/journal.pone.0160755 (PMC4993490; doi:10.1371/journal.pone.0160755)
Supplement: S3 Table — (DOCX) [file pone.0160755.s005.docx]

**S3 Table. Classification of downregulated genes (RNA-Seq data) based on biological process, molecular function and cell component**

Category and Term Genes  *P value*

| \| \| **Go for biological process** \|  \| \|  \| \| --- \| --- \| --- \| --- \| \| GO:0008152~metabolic process \| \| *Abcc9,E,Atp2a2,Pde1c,,Atp1a2,Acss1* \| 0.000006 \| \| GO:0016311~dephosphorylation \| \| *Ptpn3,Fbp2,Nt5c1a,Pfkfb1,Dusp7* \| 0.025918 \| \| GO:0016310~phosphorylation \| \| *Mylk4,Ephb1,Stk39,Ip6k3,Pfkfb1* \| 0.026211 \| \| GO:0006810~transport \| \| *Atp2a2,Cacna1s,Aqp7,Ryr2,Atp1a2* \| 0.000901 \| \| GO:0006813~potassium ion transport \| \| *Abcc9,Kcnn1,Atp1a2,Kcnj5,Kcne1* \| 0.000530 \| \| GO:0034765~regulation of ion transmembrane transport \| \| *Cacna1s,Kcnip2,Scn4a,Kcnj5,Kcne1* \| 0.001358 \| \| GO:0071805~potassium ion transmembrane transport \| \| *Abcc9,Abcc8,Kcnn1,Atp1a2,Kcnj5* \| 0.000003 \| \| GO:0055114~oxidation-reduction process \| \| *Steap3,Adhfe1,,Cyp26b1,Scd4,Acadm* \| 0.001054 \| \| GO:0006631~fatty acid metabolic process \| \| *Acot11,Ppara,Gpam,Acsm5,Acadm* \| 0.011510 \| \| GO:0019432~triglyceride biosynthetic process \| \| *Lpl,Gpam* \| 0.029800 \| \| GO:0006826~iron ion transport \| \| *Steap3,Slc40a1* \| 0.047416 \| \| GO:0006754~ATP biosynthetic process \| \| *Atp2a2,Slc25a13,Atp1a2* \| 0.025843 \| \| GO:0002026~regulation of the force of heart contraction \| \| *Atp2a2,Pln,Atp1a2* \| 0.010792 \| \| GO:0043268~positive regulation of potassium ion transport \| \| *Stk39,Abcc8* \| 0.025395 \| \| GO:0045822~negative regulation of heart contraction \| \| *Atp2a2,Pln,Atp1a2* \| 0.001671 \| \| GO:0009058~biosynthetic process \| \| *Gal3st3,Gpt,Aldh1l2,Gpt2* \| 0.012704 \| \| GO:0008016~regulation of heart contraction \| \| *Hrc,Gja1,Nos2,Hopx* \| 0.001053 \| \| GO:0051481~reduction of cytosolic calcium ion concentration \| \| *Drd2,Atp1a2* \| 0.021760 \| \| GO:0071222~cellular response to lipopolysaccharide \| \| *Cmpk2,Gbp2,Nos2* \| 0.024309 \| \| GO:0002070~epithelial cell maturation \| \| *Gja1,Kcne1* \| 0.018102 \| \| GO:0000266~mitochondrial fission \| \| *Mtfp1,Lpin1* \| 0.021760 \| \| GO:0010107~potassium ion import \| \| *Abcc9,Kcnj11* \| 0.015942 \| \| **Go for molecular function** \| \|  \|  \| \| GO:0016787~hydrolase activity \| \| *Acot11,Atp2a2,Fbp2,Lpl,Pde1c,Atp1a2* \| 0.000161 \| \| GO:0008092~cytoskeletal protein binding \| \| *Epb4.1l4a,Ptpn3,Epb4.1l3* \| 0.012816 \| \| GO:0016740~transferase activity \| \| *Ephb1,Stk39,Gpt,Gsta4,Epha4,Pfkfb1* \| 0.030057 \| \| GO:0003824~catalytic activity \| \| *Echdc3,Atp2a2,Lpl,Pde1c,Atp1a2,Pfkfb1* \| 0.014677 \| \| GO:0005524~ATP binding \| \| *Abcc9,Atp2a2,Atp1a2,Pfkfb1,Acss1,Wnk2* \| 0.032746 \| \| GO:0016301~kinase activity \| \| *Mylk4,Ephb1,Stk39,Sh3kbp1,Stk10,Wnk2* \| 0.000001 \| \| GO:0000166~nucleotide binding \| \| *Mylk4,Atp2a2,Atp1a2,Ppargc1b,Acss1* \| 0.016757 \| \| GO:0005096~GTPase activator activity \| \| *Tbc1d10c,Plxnb1,Rap1gap2,Rgs2* \| 0.048320 \| \| GO:0005216~ion channel activity \| \| *Scn4b,Cacna1s,Kcnj11,Kcnn1,Ryr2* \| 0.041455 \| \| GO:0005244~voltage-gated ion channel activity \| \| *Cacna1s,Kcnip2,Scn4a,Kcnj5,Kcne1* \| 0.000008 \| \| GO:0005267~potassium channel activity \| \| Abcc9,Abcc8,Kcnip2,Kcne1 \| 0.000134 \| \| GO:0016491~oxidoreductase activity \| \| *Steap3,Scd4,Rsad1,Nos2,Acadm* \| 0.002761 \| \| GO:0019904~protein domain specific binding \| \| *Whrn,Ppara,Mlf1,Wnt5a,Clec3b,* \| 0.000904 \| \| GO:0035255~ionotropic glutamate receptor binding \| \| *Drd2,Myo5b* \| 0.009119 \| \| GO:0004091~carboxylesterase activity \| \| *Acot11,Siae,Ces1d* \| 0.038697 \| \| GO:0005509~calcium ion binding \| \| *Atp2a2,,Syt7,Egf,Slc25a13,Calr3* \| 0.035784 \| \| GO:0046966~thyroid hormone receptor binding \| \| *Med1,Nr0b2* \| 0.045769 \| \| GO:0005102~receptor binding \| \| *Drd2,Lgi1,Pla2g5,Lpl,Wnt5a,Gja1* \| 0.014552 \| \| GO:0048155~S100 alpha binding \| \| *Atp2a2,Fgf1* \| 0.032775 \| \| GO:0051219~phosphoprotein binding \| \| *Grb14,Dpysl4,Fkbp4* \| 0.007856 \| \| GO:0008201~heparin binding \| \| *Fgf1,Pla2g5,Lpl,Clec3b* \| 0.045641 \| \| GO:0030170~pyridoxal phosphate binding \| \| *Gpt,Gpt2,Alb* \| 0.047614 \| \| GO:0005104~fibroblast growth factor receptor binding \| \| *Fgf1,Fgf16* \| 0.041886 \| \| GO:0005242~inward rectifier potassium channel activity \| \| *Kcnj11,Kcnj5,Kcnj3* \| 0.005409 \| \| GO:0008483~transaminase activity \| \| *Gpt,Gpt2* \| 0.003101 \| \| GO:0046875~ephrin receptor binding \| \| *Efnb3,Epha4* \| 0.007856 \| \| GO:0019902~phosphatase binding \| \| *Chchd3,Ppara,Sh2d4a* \| 0.045641 \| \| GO:0005005~transmembrane-ephrin receptor activity \| \| *Ephb1,Epha4* \| 0.035597 \| \| GO:0016769~transferase activity, transferring nitrogenous groups \| \| *Gpt,Gpt2* \| 0.010433 \| \| GO:0005272~sodium channel activity \| \| *Scn4b,Scn4a* \| 0.042459 \| \| GO:0070991~medium-chain-acyl-CoA dehydrogenase activity \| \| *Acadm* \| 0.033520 \| \| GO:0042974~retinoic acid receptor binding \| \| *Med1,Nr0b2* \| 0.045769 \| \| GO:0042975~peroxisome proliferator activated receptor binding \| \| *Lpin1,Med1,Nr0b2* \| 0.015807 \| \| GO:0005502~11-cis retinal binding \| \| *Opn4* \| 0.029879 \| \| GO:0015459~potassium channel regulator activity \| \| *Abcc9,Kcne1* \| 0.045769 \| \| GO:0050682~AF-2 domain binding \| \| *Ppargc1b* \| 0.038430 \| \| GO:0019829~cation-transporting ATPase activity \| \| *Atp1a2* \| 0.045769 \| \| GO:0004465~lipoprotein lipase activity \| \| *Lpl* \| 0.033520 \| \| GO:0015254~glycerol channel activity \| \| *Aqp7* \| 0.033520 \| \| GO:0030339~fatty-acyl-ethyl-ester synthase activity \| \| *Ces1d* \| 0.045769 \| \| GO:0005280~hydrogen:amino acid symporter activity \| \| *Slc36a2* \| 0.045769 \| \| GO:0005115~receptor tyrosine kinase-like orphan receptor binding \| \| *Wnt5a* \| 0.033520 \| \| GO:0017129~triglyceride binding \| \| *Lpl* \| 0.033520 \| \| GO:0036033~mediator complex binding \| \| *Med1* \| 0.033520 \| \| **Go for Cell component** \| \|  \|  \| \| GO:0005634~nucleus \| \| *Ppara,Fgf1,Foxo6,Med1,Ppargc1b* \| 0.000001 \| \| GO:0030054~cell junction \| \| *Lgi1,Ppl,Chrna2,Epha4,Lrrc4b,Gja1* \| 0.016604 \| \| GO:0005759~mitochondrial matrix \| \| *Ppif,Gstk1,Acss1,Acadm* \| 0.027889 \| \| GO:0005743~mitochondrial inner membrane \| \| *Mtfp1,Gstk1,Dusp18,Bckdhb* \| 0.003962 \| \| GO:0005515~protein binding \| \| Ppara,Med1,Egf,Lpl,Wnt5a,PNos2 \| 0.002313 \| \| \| --- \| --- \| --- \| --- \| --- \| --- \| --- \| --- \| --- \| --- \| --- \| --- \| --- \| --- \| --- \| --- \| --- \| --- \| --- \| --- \| --- \| --- \| --- \| --- \| --- \| --- \| --- \| --- \| --- \| --- \| --- \| --- \| --- \| --- \| --- \| --- \| --- \| --- \| --- \| --- \| --- \| --- \| --- \| --- \| --- \| --- \| --- \| --- \| --- \| --- \| --- \| --- \| --- \| --- \| --- \| --- \| --- \| --- \| --- \| --- \| --- \| --- \| --- \| --- \| --- \| --- \| --- \| --- \| --- \| --- \| --- \| --- \| --- \| --- \| --- \| --- \| --- \| --- \| --- \| --- \| --- \| --- \| --- \| --- \| --- \| --- \| --- \| --- \| --- \| --- \| --- \| --- \| --- \| --- \| --- \| --- \| --- \| --- \| --- \| --- \| --- \| --- \| --- \| --- \| --- \| --- \| --- \| --- \| --- \| --- \| --- \| --- \| --- \| --- \| --- \| --- \| --- \| --- \| --- \| --- \| --- \| --- \| --- \| --- \| --- \| --- \| --- \| --- \| --- \| --- \| --- \| --- \| --- \| --- \| --- \| --- \| --- \| --- \| --- \| --- \| --- \| --- \| --- \| --- \| --- \| --- \| --- \| --- \| --- \| --- \| --- \| --- \| --- \| --- \| --- \| --- \| --- \| --- \| --- \| --- \| --- \| --- \| --- \| --- \| --- \| --- \| --- \| --- \| --- \| --- \| --- \| --- \| --- \| --- \| --- \| --- \| --- \| --- \| --- \| --- \| --- \| --- \| --- \| --- \| --- \| --- \| --- \| --- \| --- \| --- \| --- \| --- \| --- \| --- \| --- \| --- \| --- \| --- \| --- \| --- \| --- \| --- \| --- \| --- \| --- \| --- \| --- \| --- \| --- \| --- \| --- \| --- \| --- \| --- \| --- \| --- \| --- \| --- \| --- \| --- \| --- \| --- \| --- \| --- \| --- \| --- \| --- \| --- \| --- \| --- \| --- \| --- \| --- \| --- \| --- \| --- \| --- \| --- \| --- \| --- \| --- \| --- \| --- \| --- \| --- \| --- \| --- \| --- \| --- \| --- \| --- \| --- \| --- \| --- \| --- \| --- \| --- \| --- \| --- \| --- \| --- \| --- \| --- \| --- \| --- \| --- \| --- \| --- \| --- \| --- \| --- \| --- \| --- \| --- \| --- \| --- \| --- \| --- \| --- \| --- \| --- \| --- \| --- \| --- \| --- \| --- \| --- \| --- \| --- \| --- \| --- \| --- \| --- \| --- \| --- \| --- \| --- \| |
| --- | --- | --- | --- | --- | --- | --- | --- | --- | --- | --- | --- | --- | --- | --- | --- | --- | --- | --- | --- | --- | --- | --- | --- | --- | --- | --- | --- | --- | --- | --- | --- | --- | --- | --- | --- | --- | --- | --- | --- | --- | --- | --- | --- | --- | --- | --- | --- | --- | --- | --- | --- | --- | --- | --- | --- | --- | --- | --- | --- | --- | --- | --- | --- | --- | --- | --- | --- | --- | --- | --- | --- | --- | --- | --- | --- | --- | --- | --- | --- | --- | --- | --- | --- | --- | --- | --- | --- | --- | --- | --- | --- | --- | --- | --- | --- | --- | --- | --- | --- | --- | --- | --- | --- | --- | --- | --- | --- | --- | --- | --- | --- | --- | --- | --- | --- | --- | --- | --- | --- | --- | --- | --- | --- | --- | --- | --- | --- | --- | --- | --- | --- | --- | --- | --- | --- | --- | --- | --- | --- | --- | --- | --- | --- | --- | --- | --- | --- | --- | --- | --- | --- | --- | --- | --- | --- | --- | --- | --- | --- | --- | --- | --- | --- | --- | --- | --- | --- | --- | --- | --- | --- | --- | --- | --- | --- | --- | --- | --- | --- | --- | --- | --- | --- | --- | --- | --- | --- | --- | --- | --- | --- | --- | --- | --- | --- | --- | --- | --- | --- | --- | --- | --- | --- | --- | --- | --- | --- | --- | --- | --- | --- | --- | --- | --- | --- | --- | --- | --- | --- | --- | --- | --- | --- | --- | --- | --- | --- | --- | --- | --- | --- | --- | --- | --- | --- | --- | --- | --- | --- | --- | --- | --- | --- | --- | --- | --- | --- | --- | --- | --- | --- | --- | --- | --- | --- | --- | --- | --- | --- | --- | --- | --- | --- | --- | --- | --- | --- | --- | --- | --- | --- | --- | --- | --- | --- | --- | --- | --- | --- | --- | --- | --- | --- | --- | --- | --- | --- | --- | --- | --- | --- | --- | --- | --- | --- | --- | --- |
