# Supplementary material for: Cardiomyocyte-Specific Ablation of Med1 Subunit of the Mediator Complex Causes Lethal Dilated Cardiomyopathy in Mice
Source: PLoS One. 2016 Aug 22;11(8):e0160755. doi: 10.1371/journal.pone.0160755 (PMC4993490; doi:10.1371/journal.pone.0160755)
Supplement: S5 Table — (DOCX) [file pone.0160755.s007.docx]

**S5 Table. Classification of upregulated genes (RNA-Seq data) based on biological process, molecular function and cell component**

Category and Term Genes *P value*

| \| \| **Go for biological process** \|  \| \|  \| \| --- \| --- \| --- \| --- \| \| \| GO:0043410 \| positive regulation of MAPK cascade \| *Itgav,Sorbs3,Igf1r* \| 0.0052985 \| \| --- \| --- \| --- \| --- \| \| GO:0006811 \| ion transport \| *Mlc1,Cacnb1,Slc17a7,Chrnb1* \| 0.0054956 \| \| GO:0006950 \| response to stress \| *Hsp90aa1,Hspa1,Ppp1r15a,Adm,Uchl1* \| 1.561E-07 \| \| GO:0006917 \| induction of apoptosis \| *Tgfb2,Trp53inp1,Thbs1,Tnfrsf12a,Phlda3* \| 0.0001487 \| \| GO:0030199 \| collagen fibril organization \| *Tgfb2,Col5a2* \| 0.0003269 \| \| GO:0045766 \| positive regulation of angiogenesis \| *Cx3cl1,Thbs1,Serpine1,Sphk1,Adm* \| 2.12E-05 \| \| GO:0002026 \| regulation of the force of heart contraction \| *Adm,Myl4* \| 0.0033376 \| \| GO:0042326 \| negative regulation of phosphorylation \| *Cdkn1a,Ahsg* \| 0.0097372 \| \| GO:0051496 \| positive regulation of stress fiber assembly \| *Sorbs3,Ctgf* \| 0.0045379 \| \| GO:0060047 \| heart contraction \| *Gpx1,Ace* \| 0.0006304 \| \| GO:0042744 \| hydrogen peroxide catabolic process \| *Gpx3,Gpx1* \| 0.0023123 \| \| GO:0000302 \| response to reactive oxygen species \| *Gpx1,Ptprn* \| 0.0023123 \| \| GO:0071578 \| zinc ion import \| *Slc39a6* \| 0.009584 \| \| GO:0006937 \| regulation of muscle contraction \| *Casq1,Tnni1* \| 0.0037185 \| \| GO:0060401 \| cytosolic calcium ion transport \| *Ctgf* \| 0.0048035 \| \| GO:0014809 \| regulation of skeletal muscle contraction \| *Casq1* \| 0.0048035 \| \| GO:0051282 \| regulation of sequestering of calcium ion \| *Casq1* \| 0.0048035 \| \| GO:0051280 \| negative regulation of calcium ion \| *Tgfb2* \| 0.009584 \| \| GO:0006982 \| response to lipid hydroperoxide \| *Gpx1* \| 0.009584 \| \| GO:0043251 \| sodium-dependent organic anion transport \| *Slc10a6* \| 0.0048035 \| \| GO:2000425 \| regulation of apoptotic cell clearance \| *Itgav* \| 0.0048035 \|   **Go for molecular function**   \| GO:0005515 \| protein binding (MF) \| *Cdkn1a,Tpm2,Hsp90aa1,Cacnb1,Hspa1a* \| 4.1065E-12 \| \| --- \| --- \| --- \| --- \| \| GO:0005520 \| insulin-like growth factor binding (MF) \| *Wisp2,Igf1r* \| 0.00371848 \| \| GO:0005158 \| insulin receptor binding (MF) \| *Dok5,Igf1r* \| 0.00973715 \| \| GO:0008083 \| growth factor activity (MF) \| *Gdf15,Tgfb2,Ctgf,Inhbb,Wisp2* \| 0.000578671 \| \| GO:0005215 \| transporter activity (MF) \| *Rbp1,Apod,Sypl2,Lcn2,Doc2b* \| 0.00226308 \| \| GO:0008237 \| metallopeptidase activity (MF) \| *Mmp3,Dpep1,Mmp23,Adamtsl2,Ace* \| 0.000791985 \| \| GO:0002020 \| protease binding (MF) \| *Panx1,Lcn2,Serpine1,Hspa1b* \| 0.000178428 \| \| GO:0005509 \| calcium ion binding (MF) \| *Mmp3,Casq1,Actn1,Fbn2* \| 1.26245E-06 \| \| GO:0005178 \| integrin binding (MF) \| *Thbs1,Thbs4,Ctgf,Actn1,Gpnmb* \| 1.97389E-05 \| \| GO:0001968 \| fibronectin binding (MF) \| *Thbs1,Thbs4,Ctgf* \| 0.000136028 \| \| GO:0005544 \| calcium-dependent phospholipid binding (MF) \| *Anxa8,Doc2b* \| 0.00691325 \| \| GO:0008307 \| structural constituent of muscle (MF) \| *Synm,Pdlim3,Krt19* \| 0.000070539 \| \| GO:0050840 \| extracellular matrix binding (MF) \| *Eln,Smoc1,Fbln2,Thbs1,Bgn* \| 1.97047E-08 \| \| GO:0004586 \| ornithine decarboxylase activity (MF) \| *Adc* \| 0.00958402 \| \| GO:0008792 \| arginine decarboxylase activity (MF) \| *Adc* \| 0.00480348 \| \| GO:0031700 \| adrenomedullin receptor binding (MF) \| *Adm* \| 0.00480348 \| \| GO:0008481 \| sphinganine kinase activity (MF) \| *Sphk1* \| 0.00958402 \| \| GO:0097024 \| protein kinase C alpha binding (MF) \| *Itgav* \| 0.00958402 \| \| GO:0002135 \| CTP binding (MF) \| *Hsp90aa1* \| 0.00958402 \| \| GO:0055077 \| gap junction hemi-channel activity (MF) \| *Panx1* \| 0.00480348 \| \| \|  \|  \| \| **Go for Cell component** \| \|  \|  \| \| \| --- \| --- \| --- \| --- \| --- \| --- \| --- \| --- \| --- \| --- \| --- \| --- \| --- \| --- \| --- \| --- \| --- \| --- \| --- \| --- \| --- \| --- \| --- \| --- \| --- \| --- \| --- \| --- \| --- \| --- \| --- \| --- \| --- \| --- \| --- \| --- \| --- \| --- \| --- \| --- \| --- \| --- \| --- \| --- \| --- \| --- \| --- \| --- \| --- \| --- \| --- \| --- \| --- \| --- \| --- \| --- \| --- \| --- \| --- \| --- \| --- \| --- \| --- \| --- \| --- \| --- \| --- \| --- \| --- \| --- \| --- \| --- \| --- \| --- \| --- \| --- \| --- \| --- \| --- \| --- \| --- \| --- \| --- \| --- \| --- \| --- \| --- \| --- \| --- \| --- \| --- \| --- \| --- \| --- \| --- \| --- \| --- \| --- \| --- \| --- \| --- \| --- \| --- \| --- \| --- \| --- \| --- \| --- \| --- \| --- \| --- \| --- \| --- \| --- \| --- \| --- \| --- \| --- \| --- \| --- \| --- \| --- \| --- \| --- \| --- \| --- \| --- \| --- \| --- \| --- \| --- \| --- \| --- \| --- \| --- \| --- \| --- \| --- \| --- \| --- \| --- \| --- \| --- \| --- \| --- \| --- \| --- \| --- \| --- \| --- \| --- \| --- \| --- \| --- \| --- \| --- \| --- \| --- \| --- \| --- \| --- \| --- \| --- \| --- \| --- \| --- \| --- \| --- \| --- \| --- \| --- \| --- \| --- \| --- \| --- \| --- \| --- \|      \| GO:0005829 \| cytosol \| *Itgav,Cdkn1a,Hsp90aa1,Lcn2,Hspa1b* \| 0.00428874 \| \| --- \| --- \| --- \| --- \| \| GO:0005634 \| nucleus \| *Mmp3,Trp53inp1,Atf3,Csrp1,Kif1a* \| 0.0052987 \| \| GO:0005576 \| extracellular region \| *Mmp3Tgfb2,,Wisp2,Fmod,Fbn2* \| 3.6841E-26 \| \| GO:0048471 \| perinuclear region of cytoplasm \| *Nppa,Nppb,Cx3cl1,Ctgf,Hspa1b* \| 0.00121833 \| \| GO:0005622 \| intracellular \| *Itgav,Hsp90aa1,Cacnb1,Igf1r* \| 0.00787355 \| \| GO:0005783 \| endoplasmic reticulum \| *Panx1,Qsox1,Jsrp1,Slc39a6* \| 0.00221906 \| \| GO:0042383 \| sarcolemma \| *Casq1,Bgn,Krt19* \| 0.0039949 \| \| GO:0005581 \| collagen \| *Col8a2,Emid2,Col8a1,Col12a1* \| 1.3534E-07 \| \| GO:0016529 \| sarcoplasmic reticulum \| *Jsrp1,Cacnb1,Casq1* \| 0.0010903 \| \| GO:0031012 \| extracellular matrix \| *Mmp3,Tgfb2,Hsp90aa1,Ahsg* \| 1.81941E-08 \| \| GO:0001725 \| stress fiber \| *Myl9,Pdlim7,Actn1,Zyx,Acta1* \| 4.30236E-06 \| \| GO:0008305 \| integrin complex \| *Itgav,Itgbl1* \| 0.00590799 \| \| GO:0042629 \| mast cell granule \| *Nppa* \| 0.0143417 \| \| GO:0044420 \| extracellular matrix part \| *Cilp* \| 0.00480348 \| \| GO:0032127 \| dense core granule membrane \| *Actn1* \| 0.0143417 \| \| GO:0033018 \| sarcoplasmic reticulum lumen \| *Casq1* \| 0.00480348 \| |
| --- | --- | --- | --- | --- | --- | --- | --- | --- | --- | --- | --- | --- | --- | --- | --- | --- | --- | --- | --- | --- | --- | --- | --- | --- | --- | --- | --- | --- | --- | --- | --- | --- | --- | --- | --- | --- | --- | --- | --- | --- | --- | --- | --- | --- | --- | --- | --- | --- | --- | --- | --- | --- | --- | --- | --- | --- | --- | --- | --- | --- | --- | --- | --- | --- | --- | --- | --- | --- | --- | --- | --- | --- | --- | --- | --- | --- | --- | --- | --- | --- | --- | --- | --- | --- | --- | --- | --- | --- | --- | --- | --- | --- | --- | --- | --- | --- | --- | --- | --- | --- | --- | --- | --- | --- | --- | --- | --- | --- | --- | --- | --- | --- | --- | --- | --- | --- | --- | --- | --- | --- | --- | --- | --- | --- | --- | --- | --- | --- | --- | --- | --- | --- | --- | --- | --- | --- | --- | --- | --- | --- | --- | --- | --- | --- | --- | --- | --- | --- | --- | --- | --- | --- | --- | --- | --- | --- | --- | --- | --- | --- | --- | --- | --- | --- | --- | --- | --- | --- | --- | --- | --- | --- | --- | --- | --- | --- | --- | --- | --- | --- | --- | --- | --- | --- | --- | --- | --- | --- | --- | --- | --- | --- | --- | --- | --- | --- | --- | --- | --- | --- | --- | --- | --- | --- | --- | --- | --- | --- | --- | --- | --- | --- | --- | --- | --- | --- | --- | --- | --- | --- | --- | --- | --- | --- | --- | --- | --- | --- | --- | --- | --- | --- | --- | --- | --- | --- | --- | --- | --- | --- | --- |
